# Detecting protein complexes with multiple properties by an adaptive harmony search algorithm

**DOI:** 10.1186/s12859-022-04923-4

**Published:** 2022-10-07

**Authors:** Rongquan Wang, Caixia Wang, Huimin Ma

**Affiliations:** 1 School of Computer and Communication Engineering, University of Science and Technology Beijing, No. 30 Xueyuan Road, Haidian District, Beijing, 100083 China; 2 School of International Economics, China Foreign Affairs University, 24 Zhanlanguan Road, Xicheng District, Beijing, 100037 China

**Keywords:** Protein-protein interaction network, Protein complex, Multiple properties, Core-attachment structure, Fitness function, Adaptation harmony search algorithm

## Abstract

**Background:**

Accurate identification of protein complexes in protein-protein interaction (PPI) networks is crucial for understanding the principles of cellular organization. Most computational methods ignore the fact that proteins in a protein complex have a functional similarity and are co-localized and co-expressed at the same place and time, respectively. Meanwhile, the parameters of the current methods are specified by users, so these methods cannot effectively deal with different input PPI networks.

**Result:**

To address these issues, this study proposes a new method called MP-AHSA to detect protein complexes with Multiple Properties (MP), and an Adaptation Harmony Search Algorithm is developed to optimize the parameters of the MP algorithm. First, a weighted PPI network is constructed using functional annotations, and multiple biological properties and the Markov cluster algorithm (MCL) are used to mine protein complex cores. Then, a fitness function is defined, and a protein complex forming strategy is designed to detect attachment proteins and form protein complexes. Next, a protein complex filtering strategy is formulated to filter out the protein complexes. Finally, an adaptation harmony search algorithm is developed to determine the MP algorithm’s parameters automatically.

**Conclusions:**

Experimental results show that the proposed MP-AHSA method outperforms 14 state-of-the-art methods for identifying protein complexes. Also, the functional enrichment analyses reveal that the protein complexes identified by the MP-AHSA algorithm have significant biological relevance.

**Supplementary Information:**

The online version contains supplementary material available at 10.1186/s12859-022-04923-4.

## Background

Upon completing the human genome project, proteomics has become the focus in the post-genomic era. Proteins do not function only as single units. Instead, they form protein-protein interaction (PPI) networks and/or functional protein complexes [1]. Since most biological cellular processes are performed by protein complexes [2], identifying these operating units is an essential step for studying cells. Many experimental methods that can produce high-throughput PPI data have been proposed to identify protein complexes within living cells, e.g., tandem affinity purification with mass spectrometry (TAP-MS) [3]. However, the existing experimental methods are expensive and time-consuming and may result in false-positive, or false-negative results [4].

**Fig. 1:**
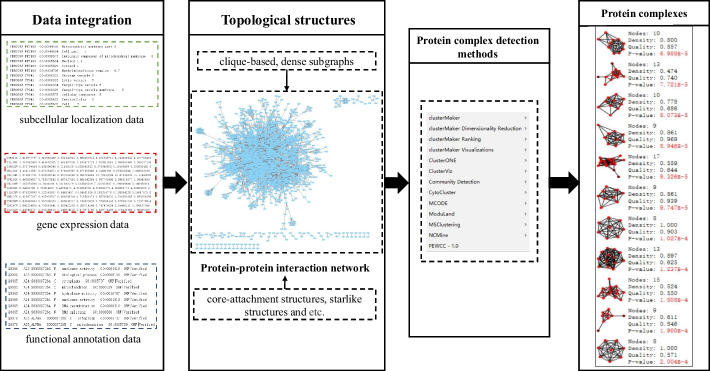
The detection process of protein complexes from a protein-protein interaction (PPI) network

Genome-scale PPI data can be obtained through high-throughput approaches, such as yeast-two-hybrid [5]. These PPI data can be formulated as an undirected graph in which the nodes and edges correspond to proteins and pairwise interactions. Meanwhile, most proteins are highly interactive with proteins in the same protein complex, which allows them to perform biological functions. Hence, the dense region in a PPI network can be identified as a protein complex. Thus, detecting protein complexes is similar to identifying communities in complex networks [6]. Based on this, the problem of identifying protein complexes is usually transformed into the issue of soft graph clustering. Figure 1 shows the detection process of protein complexes from a PPI network.

### Related work

Over the past decade, various computational methods have been proposed to identify protein complexes in PPI networks automatically [7]. Among them, IPCA [8], and SPICi [9] identify local dense subgraphs as local protein complexes instead of globally clustering a network based on different network properties and concepts [10]. By contrast, other methods, such as MCL [11], and RRW [12], apply random walks, which is a classic global protein complex identification approach. These methods mine global protein complexes by manipulating the network nodes’ transition probabilities or stochastic flows. In particular, RNSC [13] identifies global protein complexes by efficiently separating networks into clusters using a cost function. To detect sparse protein complexes, PC2P [14] has been proposed to mine protein complexes as biclique spanned subgraphs (including both sparse and dense subgraphs) using the network partitioning method. Besides, other methods such as CMC [15] identify protein complexes by merging, mixing, or deleting different types of cliques or k-cores, COACH [16] and WPNCA [17] take the core-attachment structure into account to detect protein complexes. In addition, some methods, such as OH-PIN [18], detect protein complexes using hierarchical clustering algorithms based on similarity or distance. Moreover, several methods, such as ClusterONE [19], and SE-DMTG [20], start from a protein or edge and expand it using a greedy algorithm for detecting protein complexes. Recently, with the increasing research on swarm intelligence optimization algorithms, many study methods have transformed the protein complex identification into an optimization problem [21, 22]. However, these methods have two limitations: they only identify protein complexes with a single topological structure, and they cannot automatically and correctly set the input parameters of the algorithm according to different input datasets.

High-throughput experiment-derived PPIs may have false-positive or false-negative results, significantly affecting protein complex identification. Therefore, some computational methods, such as PEWCC [23] and EWCA [24], have been developed to improve the accuracy of protein complex identification by using the topology of PPI networks. Meanwhile, to reduce the effect of both false-positive and false-negative interactions on the performance of protein complex detection methods, GCC-v [25] and CUBCO [26] are designed to predict protein complexes by scoring and incorporating missing interactions. Experiment results show their performance outperformed other state-of-the-art approaches across different species.

Furthermore, some other methods attempt to integrate biological properties. For instance, WEC [27] uses gene expression data to detect highly interconnected and co-expressed protein complexes, whereas CPredictor5.0 [28] integrates gene ontology (GO) data and topological information of PPIs. Some methods [29, 30] use subcellular localization data to identify protein complexes. Recently, idenPC-MIIP [31] has been proposed to identify protein complexes based on the relationship of important mutually-interacting partners. Additionally, Wu et al. [32] developed idenPC-CAP to identify protein complexes from RNA-protein heterogeneous interaction networks. Most of the above methods cannot reflect the dynamic characteristic of protein complexes [33] because the PPI network will change over time and depends on its surrounding conditions. Therefore, current methods have considered dynamic cellular systems to create dynamic PPIs by using time-course gene expression data [34]. For example, based on the 3-sigma principle, some methods [35, 36] identify the active points of a protein in a time-serial gene expression data and to generate a series of time-sequenced subnetworks for identifying dynamic protein complexes. However, these methods usually identify many small false-positive protein complexes. In summary, using different types of biological properties or data to compensate for the PPI networks can improve the accuracy of PPI network-based detection of protein complexes.

In recent years, some supervised learning methods have been developed, including ClusterEPs [37] and ClusterSS [38], to identify protein complexes by using the properties of known protein complexes. In 2021, Zaki et al. [39] introduced graph convolutional network approaches to improve the ability to detect protein complexes. Mei et al. [40] proposed a computational framework that combines supervised learning and dense subgraph to predict protein complexes. Furthermore, Liu et al. [41] proposed a new algorithm based on a semi-supervised model to identify significant protein complexes with clear module structures. Additionally, ELF-DPC [42] is an ensemble learning framework for detecting protein complexes based on structural modularity and a trained voting regressor model. However, the performance of these methods is limited by the training data size. With more known protein complexes available, detecting protein complexes by supervised learning methods will be further explored.

### Motivation

Some researchers have illustrated that a protein complex with a core-attachment structure consists of two parts: a protein complex core and attachment proteins [43]. Various methods based on core-attachment structure have proposed to detect protein complexes, such as MCL-CA [44], CACHET [45], COACH [16], Ma [46], WPNCA [17]. However, these methods ignore that the core proteins in the protein complex core are often co-localized, co-expressed, and have similar functions [30, 47], form the main functional part of the protein complex. Meanwhile, attachment proteins bind to the proteins of the protein complex core, helping to perform their functions. Current studies [7] classify protein complexes into global [11, 13] and local protein complexes [8, 20]. Local protein complexes are protein complexes by local-cluster-quality-based methods, and these methods identify local clusters with optimal local cluster quality in a seed growth manner. Meanwhile, global protein complexes are protein complexes by global-cluster-quality-based methods, and they search for an optimal clustering result with the best global-cluster-quality function value. This paper designs a local protein complex core detection strategy to mine local protein complex cores and form local protein complexes. The MCL method identifies global protein complex cores and forms global protein complexes.

However, protein complexes include both global and local protein complexes. Moreover, although various definitions of protein complexes have been proposed, most only consider single properties. Thus, a novel structural description of protein complexes considering multiple topological properties is urgently needed. Additionally, most protein complex detection methods have a common disadvantage: their parameters are specified by users, making it difficult to deal with various PPI networks effectively. In recent years, the harmony search algorithm has paid much attention in the fields of bioinformatics, such as the detection of high-order SNP epistasis and protein interactions [48], combinations, and epistasis [49–51], etc. Therefore, the improved harmony search algorithm is used to determine the parameters of the protein complex detection method in this paper. This paper will study and address the above issues.

### Our work

To overcome the disadvantages of existing methods, this paper proposes a novel approach called MP-AHSA, which combines the MP algorithm and an adaptation harmony search algorithm (AHSA) to automatically determine the parameters of the MP algorithm for the input of different PPI networks. The MP algorithm is based on the core-attachment structure and multiple properties, and it is developed to identify protein complexes in PPI networks. First, the Topological Clustering Semantic Similarity (TCSS) method [52] based on functional annotations adopted to calculate the functional similarity between two interaction proteins, and a weighted PPI network is constructed. Then, a local protein complex core detection strategy is designed based on gene expression and subcellular localization data to identify local protein complex cores. Then MCL is used to identify global protein complex cores. Next, a fitness function integrating multiple topological properties is defined to describe protein complexes. Subsequently, a new protein complex forming strategy is developed to extend global and local protein complex cores to form protein complexes. Finally, the GO annotation data is used to filter the candidate protein complexes and improve the accuracy of the protein complex detection. The experimental results show that the performance of our algorithm is better than other comparison algorithms in most cases, and the experimental results on different datasets show that our algorithm has certain robustness and stability. Furthermore, the MP-AHSA algorithm can identify protein complexes with functional significance based on the p-value. The contributions of this paper are summarized as follows:

- A fitness function is defined, and it can detect protein complexes with multiple properties;
- The MP algorithm based on the core-attachment structure is proposed, and it can detect co-localized, co-expressed protein complexes with similar functions;
- The AHSA algorithm is developed to automatically determine the parameters of the MP algorithm for the input of different PPI networks;
- The experiments on various widely used PPI networks show that the proposed MP-AHSA algorithm outperforms 14 state-of-the-art methods.

## Terminology

Herein, a PPI network is generally described as a weighted graph $G=(V, E, W)$, where *V* represents a set of proteins, *E* is a set of interactions, and *W* is a $n\times n (n=|V|)$ matrix that represents the reliability of protein pairs in the PPI network. The set of immediate neighbors of the node *v* is defined as $N(v)=\{u|(u,v)\in E, u\in V\}$. Meanwhile, we have provided a symbol table to explain these symbols in Table 1.

**Table 1:** Symbol and its explanation in this paper

ID	symbol	Explanation
1	PPI	Protein-protein interaction
2	MP	Multiple properties
3	MP-AHSA	Multiple properties and an adaptation harmony search algorithm
4	MCL	Markov cluster algorithm
5	TCSS method	Topological clustering semantic similarity
6	GO	Gene ontology
7	CC	Cellular component
8	BP	Biological process
9	MF	Molecular function
10	WCC	Weighted local clustering coefficient
11	LN(c)	The union set of the first neighbors of protein c and itself
12	V(G)	The set of proteins in G
13	SLD	Subcellular localization data
14	GED	Gene expression data
15	CEV	Co-expression threshold value
16	G	Weighted PPI network
17	GCE	Gene co-expression threshold
18	PCCs	The set of protein complex cores
19	PCC	A protein complex core
20	Neighbor(PCC)	The neighbors of protein complex core
21	cohesiveness(C)	The cohesiveness score of cluster C
22	density(C)	The weighted density of cluster C
23	awm(C)	The average weighted modularity of cluster C
24	VC	The set of proteins in the cluster C
25	EC	The set of interactions in the cluster C
26	WC	The set of weights between the protein pair in the cluster C
27	fitness(C)	The fitness function score of cluster C
28	N(PCC)	The potential attachment proteins of the cluster PCC
29	attachscore(v,PCC)	The sum of weights between protein v and the protein complex core PCC
30	CPC	A candidate protein complex
31	FPC	A filtered protein complex
32	FPCs	The set of filtered protein complexes
33	$term_{maxcommon}$	The functional annotation term with the most common proteins in the identified protein complex has
34	HAS	The harmony search algorithm
35	HMCR	The harmony memory considering rate of AHSA method
36	PAR	The pitch adjusting rate of AHSA method
37	FW	The fret width of AHSA method
38	OFfitness	The sum of the fitness function of the detected protein complexes and it is used as the objective function
39	K	The number of identified protein complexes
40	Fitness(Ci)	The fitness function of the ith identified protein complex Ci
41	i	The iteration times
42	HMs	The harmony memory
43	HM	A harmony
44	R1,R2	The variable value by randomly generated within [0,1]
45	fitnessmax,fitnessmin	The maximum and minimum values of $OF_{fitness}$ in HMs
46	HMnew	The new harmony generated
47	HMsmin	The worst harmony in HMs
48	Maxiter	The termination time
49	HMsbest	The best clustering HM in the harmony memory HMs
50	IPCs	The identified protein complexes

## Methods

**Fig. 2:**
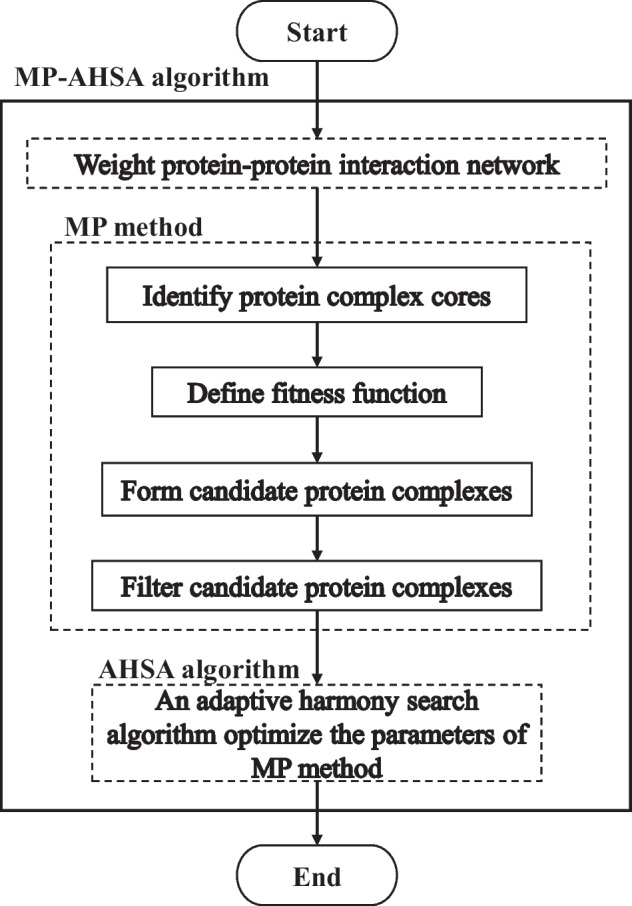
MP-AHSA algorithm detects protein complexes from PPI network


This paper proposes the MP-AHSA algorithm to identify protein complexes in PPI networks. The pipeline of the algorithm is shown in Algorithm 1, and it consists of the MP and AHSA methods. Figure 2 shows the flow of Algorithm 1. According to Algorithm 1, the MP-AHSA algorithm first constructs a weighted PPI network based on the TCSS method. Second, the MP algorithm is designed, and it first detects protein complex cores using Algorithm 2. Next, it defines a fitness function to describe protein complex in the PPI network. All protein complex cores are extended to form candidate protein complexes using fitness function and Algorithm 3. Based on the common functional annotation term, Algorithm 4 filters identified protein complexes. Finally, the AHSA algorithm is used to optimize the parameters of the MP algorithm.

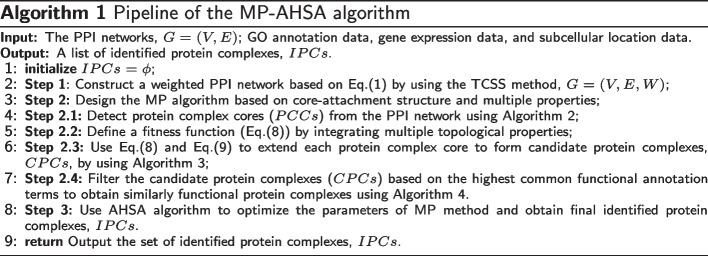


### Constructing a weighted PPI network

Recent studies [15, 24, 36] have shown that the accuracy of identifying protein complexes can be significantly improved by integrating functional annotations into a single PPI network. Therefore, this paper uses an improved algorithm, i.e., the Topological Clustering Semantic Similarity (TCSS) method (including IEA annotations) by Jain et al. [52] to calculate the semantic similarity between two interacting proteins for weighting a PPI network. In particular, this method considers an unequal depth of biological knowledge representations in different branches of the GO graph. Then, the gene annotations with GO terms downloaded from the Gene Ontology database for S.cerevisiae [53] are used to reflect the functional similarity of the proteins. According to the author’s suggestion, the topology cutoffs for the cerevisiae PPI dataset are 2.4 for CC, 3.5 for BP, and 3.3 for MF, respectively. For an edge, its semantic similarity score is calculated by using the average of the cellular component (CC), biological process (BP), and molecular function (MF) ontologies of GO by Eq. (1):

1 $$\begin{aligned} TCSS(v,u)=\frac{TCSS_{CC}(v,u)+TCSS_{BP}(v,u)+TCSS_{MF}(v,u)}{3}. \end{aligned}$$

In this way, the reliability of the PPI networks is improved based on the semantic similarity score, and a weighted PPI network is constructed.

### MP algorithm

In the following subsections, the steps of the MP algorithm are explained in detail.

#### Identifying protein complex cores

The identification of protein complex cores consists of two steps in Algorithm 2. In step 1, the initial seeds are identified, and local protein complex cores are mined based on the initial seeds. In step 2, global protein complex cores are detected by employing the MCL method [11]. Additional file 12 shows an example diagram to describe the Algorithm 2.

To detect local protein complex cores, we first introduce a weighted local clustering coefficient to detect initial seeds. Research has shown that PPI networks have a small world [54], scale-free [55], and modularity characteristics [56]. Therefore, local protein complex cores have a high local clustering coefficient [19, 36]. Thus, the higher the local clustering coefficient of the protein, the more likely the protein is to comprise the local protein complex core in the PPI network. For a protein $p_{i}$, the definition of its weighted local clustering coefficient ($WCC(p_{i})$) [57] is shown in Eq. (2):

2 $$\begin{aligned} WCC(p_{i})=\frac{2\times \sum _{(v,u)\in LN(p_{i})} w(v,u)}{\sqrt{|LN(p_{i})|}\times (|LN(p_{i})|-1)}, p_{i}\in V(G), \end{aligned}$$

where *w*(*v*, *u*) represents the weight of the edge (*v*, *u*), $|LN(p_{i})|=|\{N(p_{i})\cup \{p_{i}\} \}|$ is the number of proteins in $LN(p_{i})$, and $LN(p_{i})$ is the union set of the first neighbors of $p_{i}$ ($N(p_{i})$) and $p_{i}$, *V*(*G*) is the set of proteins in *G*.

Next, based on initial seeds, we use subcellular localization data and gene expression data to form local protein complex cores. Because some studies [2, 58] have shown that proteins in a protein complex core tend to interact with each other, and the protein complex core is generally highly co-expressed and has the same cellular localization. Thus, this paper proposes a local protein complex core identification strategy to detect local protein complex cores. Here, for subcellular localization data, *SLD*, the proteins in the same protein complex tend to have the same subcellular localization term. Second, gene expression data, *GED*, are used to estimate proteins in the same protein complex core co-expression based on the person correlation coefficient.

Generally, the gene expression data can reflect the features of proteins in a biological process under various conditions. However, for a protein, the fluctuation range of its expression is not the same. We normalize its expression value. As a result, its value is normalized using Eq. (3):

3 $$\begin{aligned} T_{i}^{'}(v)=\frac{T_{i}(v)}{\max \{T(v)\}}, \end{aligned}$$

where $T_{i}(v)$ represents the expression of protein *v* at the time point *i*, and $\max \{T(v)\}$ represents the maximum expression of protein *v* during the experimental procedure.

Furthermore, for a pair of proteins *v* and *u* in the PPI network, their gene expression profiles are denoted as $v=\{x_{1},x_{2},...,x_{n}\}$ and $u=\{y_{1},y_{2},...,y_{n}\}$, respectively. Here, the person correlation coefficient is adopted to calculate their co-expression value *CEV*(*v*, *u*) [35], and its definition is shown in Eq. (4):

4 $$\begin{aligned} CEV(v,u)=\frac{\sum _{i=1}^{n}(x_{i}-{\overline{x}})\times (y_{i}-{\overline{y}})}{\sqrt{\sum _{i=1}^{n}(x_{i}-{\overline{x}})^{2}}\times \sqrt{\sum _{i=1}^{n}(y_{i}-{\overline{y}})^{2}}}, \end{aligned}$$

where ${\overline{x}}$ and ${\overline{y}}$ represent the average of the expression of the genes encoding proteins *v* and *u* in *n* time points. To ensure that the value of *CEV*(*v*, *u*) falls within [0,1], this paper replaces *CEV*(*v*, *u*) with $CEV(v,u)=(CEV(v,u)+1)/2$. Hence, the higher the value of *CEV*(*v*, *u*), the more likely the proteins *v* and *u* to be co-expressed, and form the same protein complex.

According to weighted local clustering coefficient (Eq. (2)), subcellular localization data (two interacting proteins have the same subcellular localization term) and gene expression data and improved person correlation coefficient (Eq. (4)), we use them to identify local protein complex cores. Meanwhile, the MCL method is used to mine global protein complex cores. The pseudo-code of the mining protein complex cores is shown as Algorithm 2. First, the initial seeds are obtained, and then local protein complex cores are detected (lines 1-7). To obtain the initial seeds, the weighted clustering coefficients of each protein are calculated based on Eq. (2). All proteins are sorted in descending order based on its $WCC(p_{i})$ (lines 2-6). The top *ratio* $\%$ proteins in *V*(*G*) are selected as the initial seeds (line 7). For each protein *s* in *InitialSeeds*, if it is not visited, it is first initialized as a protein complex core, *PCC*. Meanwhile, it is marked and no longer used as a seed protein to form the protein complex core (lines 8-12). Second, the subcellular location data of the seed protein *s* (*SLD*(*s*)) are obtained. The direct neighbors of the initial protein complex core *PCC* (*Neighbor*(*PCC*)) are determined (lines 13-14). Third, for each protein $u\in Neighbor(PCC)$, if the *CEV*(*v*, *u*) of the edge between the seed protein *s* and its neighbor *u* is larger than *GCE*, and the seed protein *s* and its neighbor *u* have at least one common subcellular location term, the neighbor *u* is considered a part of the protein complex core *PCC*. It is added to the protein complex core *PCC* and marked (lines 15-23). Finally, if the protein complex core *PCC* is larger than two and does not exist in *PCCs*, it is saved (lines 24-26). The entire procedure terminates when no seed proteins need to be considered in *InitialSeeds* (lines 9-27). Then, the MCL method is employed, and its parameter *inflate* is set to detect global protein complex cores, *MCLcluster* (lines 28-29). MCL [11] is an iterative process that alternately applies two operations, i.e., expand and inflate, to mine global protein complex cores. Finally, local and global protein complex cores are combined, and redundant protein complexes are eliminated from protein complex cores, *PCCs* (lines 30-31). Note that Algorithm 2 involves three parameters: *GCE*, *inflate*, and *ratio*. This paper uses the adaptive harmony search algorithm to set the parameters automatically, as shown in Algorithm 5 (see the MP-AHSA algorithm).
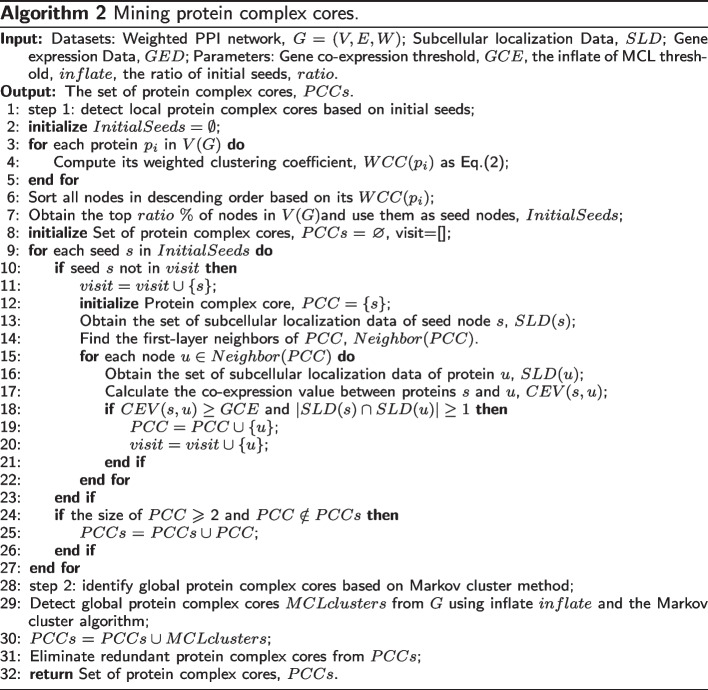


#### Fitness function

A fitness function needs to be defined to identify various topological properties of the protein complexes in the PPI network. A fitness function should combine multiple topological properties and compensate for the shortcomings of a single topological property to improve the quality of the identified protein complexes. This paper proposes a novel fitness function (Eq. (8)) by combining three topological properties including cohesiveness score (*cohesiveness*(*C*)), weighted density (*density*(*C*)), and the average weighted modularity (*awm*(*C*)) to identify protein complexes. These topological properties are defined in Eqs. (5-7):

Given the cluster, $C=(V_{C}, E_{C}, W_{C})$, where $V_{C}$ is the set of proteins in the cluster *C*, $E_{C}$ is the set of interactions in the cluster, and $W_{C}$ is the set of weights between the protein pair in the cluster. According to previous studies [19, 20], the cohesiveness score is defined in Eq. (5):

5 $$\begin{aligned} cohesiveness(C)=\frac{W_{in}(C)}{W_{in}(C)+W_{out}(C)}, \end{aligned}$$

where $W_{in}(C)$ represents the sum of weights of all edges in the cluster *C*, and $W_{out}(C)$ is the sum of weights of the edges connecting the inner proteins in *C* to other proteins in the rest of the PPI network.

According to the previously suggested hypotheses [8, 9], the higher the density of a cluster, the more likely cluster represents a protein complex. Thus, the weighted density of the cluster *C* is defined in Eq. (6):

6 $$\begin{aligned} density(C)=\frac{2\times W_{in}(C)}{|V_{C}|\times (|V_{C}|-1)}, \end{aligned}$$

where $V_{C}$ is the number of proteins in cluster *C*.

Some studies [36] have shown strong connections between the proteins in a protein complex but weak connections between the proteins outside of the protein complex. Thus, this paper proposes a new function called the average weighted modularity (awm). Awm could estimate that cluster *C* has a high average weight when connected but has a low average weight interaction with the rest of the network. awm is defined in Eq. (7):

7 $$\begin{aligned} awm(C)=\frac{AIEW(C)}{AIEW(C)+ABEW(C)}, \end{aligned}$$

The average inner edge weight ($AIEW(C)=\frac{W_{in}(C)}{|E_{C}|}$) can estimate the reliability of the internal edges of the cluster *C*, where $W_{in}(C)$ represents the sum of the weights of the edges, and $|E_{C}|$ is the number of edges in cluster *C*. Meanwhile, the average border edge weight($ABEW(C)=\frac{W_{out}(C)}{|BE_{C}|}$) can measure the reliability of the border edges in cluster *C*, where $|BE_{C}|=\{(u,v)|u \in C, v \notin C\}$ represents the number of border edges that connect cluster *C* with the rest of the PPI network.

Taking these objective functions together, this paper proposes a fitness function (*fitness*(*C*)) that combines these single objective functions to evaluate the possibility that cluster *C* is a protein complex, as shown in Eq. (8):

8 $$\begin{aligned} fitness(C)=density(C)+cohesiveness(C)+awm(C). \end{aligned}$$

Generally, a high-quality protein complex is a group of densely inter-connected but sparsely inter-connected with the rest of the PPI network. According to *fitness*(*C*), *density*(*C*) seeks a protein complex with a dense intra-connection. *cohesiveness*(*C*) and *awm*(*C*) can identify the protein complexes with densely interconnected nodes that are sparsely inter-connected to the rest of the PPI network. Therefore, this fitness function could detect various topological properties of protein complexes in PPI networks.

#### Forming protein complexes

After obtaining protein complex cores, the key is finding the attachment proteins required to form protein complexes that often surround the protein complex core. Attachment proteins are a functionally mixed group of proteins that assist the protein complex core in performing subordinate functions [6, 24, 36]. Meanwhile, attachment proteins directly and closely interact with their protein complex core. Additional file 13 shows an example diagram to describe the Algorithm 3.

Given a protein complex core *PCC* in the PPI network, all its neighbor proteins can be considered potential attachment proteins, *N*(*PCC*). All its inner proteins are removed from the current protein complex core *CPC*. For an attachment protein $v\in N(PCC)$, this paper defines *attachscore*(*v*, *PCC*) between the potential attachment protein *v* and the protein complex core *PCC* in the PPI network according to Eq. (9).

9 $$\begin{aligned} attachscore(v,PCC)=\frac{\sum _{v\notin PCC,u\in PCC}w(v,u)}{|PCC|}, \end{aligned}$$

where $\sum _{v\notin PCC,u\in PCC}w(v,u)$ represents the sum weight of the potential attachment protein *v* that connects with the protein complex core *PCC*, and |*PCC*| is the number of proteins in the protein complex core *PCC*. Thus, *attachscore*(*v*, *PCC*) can effectively estimate the interaction tightness between the potential attachment protein *v* and the protein complex core *PCC* in the PPI network.

The pseudo-code of the method for obtaining protein complexes is shown in Algorithm 3. For each protein complex core in Algorithm 3, the main operation is to iteratively add its neighbor nodes and delete its internal nodes to identify protein complexes. It includes two steps: Step 1 inserts neighbors into the current protein complex core (lines 5-16). Step 2 deletes inner nodes from the current protein complex core (lines 17-27). Finally, because the diameter of protein complexes is 2, we set the termination condition of the above two-step iteration as that the iteration number is greater than or equal to 2 or the current protein complex is no longer changed (lines 3, 28-29). For current protein complex core, *PCC* (line 1), we first initialize a candidate protein complex *CPC*, the number of iterations *count*, and Iteration termination mark *mark*. Next, we form a candidate protein complex by detecting its attachment proteins based on *attachscore*(*v*, *CPC*) (Eq. (9)) and *fitness*(*CPC*) (Eq. (8)). The direct neighboring proteins of the protein complex core, *N*(*CPC*), are obtained (line 2). For example, we first do is that the attachment proteins are added into its protein complex core to form the protein complex. If the size of the protein complex core *N*(*CPC*) is larger than or equal to 2 or adjust == 1 (line 7), for each potential attachment protein $w\in N(CPC)$, the potential attachment protein $node_{max}$ with the largest *attachscore*(*w*, *CPC*) with the protein complex core *CPC* is selected as a candidate attachment protein (line 8). Then, the fitness of $CPC\cup \{node_{max}\}$, and the fitness of *CPC* are calculated using Eq. (9), and if the potential attachment protein $node_{max}$ is inserted into *CPC*, and the fitness of current protein complex core can be increased. The potential attachment protein $node_{max}$ is inserted into *CPC* to increase the *fitness*(*CPC*) (Eq. (8)) of *CPC* (lines 11) and protein $node_{max}$ is removed from *N*(*CPC*). This process is performed iteratively, and once the new attachment protein $node_{max}$ is inserted into the protein complex core *CPC*, the protein complex core *CPC* is updated. That is, the neighbors of the new cluster *CPC* are re-constructed, and the potential attachment protein $node_{max}\in N(CPC)$ with the largest *attachscore*(*v*, *CPC*) and the neighbors of the new protein complex core *CPC* are re-calculated. Also, the algorithm is redirected to the new protein complex core *CPC* (lines 8-15). Otherwise, this process is terminated (lines 13-15). Next, we obtain the inner nodes based on *CPC*, then we find the $node_{min}$ in *I*(*CPC*) having the minimum value of $attachscore(v,CPC- \{node_{min})$ according to Eq. (9) and calculate the fitness of $CPC- \{node_{min}\}$ and the fitness of *CPC* based on Eq. (8). If the inner node $node_{min}$ is deleted from current protein complex *CPC* can increase the fitness of *CPC*, and the inner node $node_{min}$ is removed from *CPC*. This process is performed iteratively until $|I(CPC)|< 4$ and adjust == 0. Steps 1 and 2 are executed circularly until the number of iterations exceeds two or the current protein complex is no longer changed (lines 28-30). For the current protein complex core *CPC*, if its *fitness*(*CPC*) is larger than 0, and its size is larger than or equal to 3, the detected protein complex is inserted into identified protein complexes (*CPCs*) (lines 32-35). These steps are repeated until the protein complex cores (*CPCs*) are empty (lines 1-36). Considering that some protein complexes may be the same, this paper eliminates these redundant protein complexes (line 37). This way, the candidate protein complex set *CPCs* is generated (line 38).
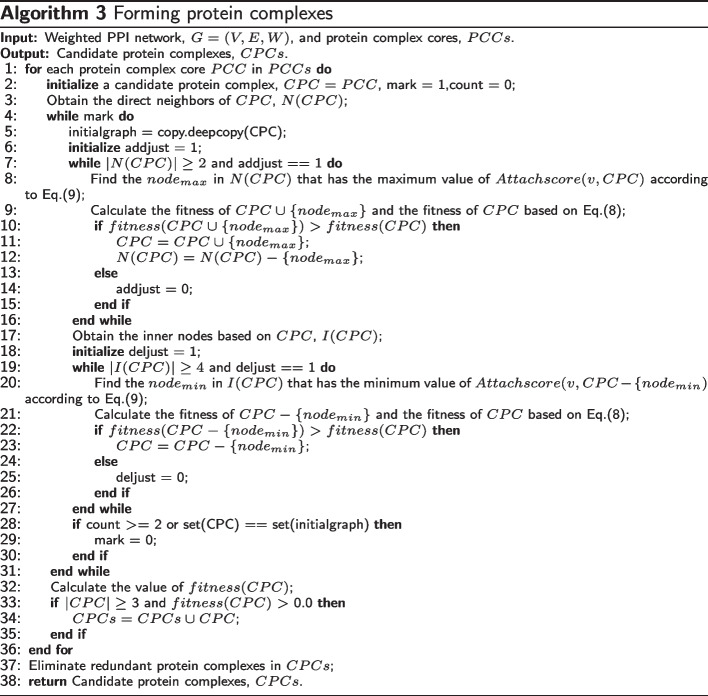


#### Filtering candidate protein complexes

Functional annotations are used to filter the detected protein complexes. The post-processing subroutine for filtering identified protein complexes is shown in Algorithm 4. Based on functional annotations, for each candidate protein complex, *CPC* in the identified candidate protein complexes *CPCs*, a filtered protein complex *FPC* is first initialized (line 1). Then the functional annotation term with the most common proteins in the candidate protein complex is determined, $term_{maxcommon}$ (lines 3-4). Next, for each protein *u* in the candidate protein complex *CPC*, the set of functional annotation terms, i.e., *FAT*(*u*), is obtained (lines 5-6). If the protein *u* has $term_{maxcommon}$, the protein *u* is added to the filtered protein complex *FPC* (lines 7-9). This process is continued until all proteins in the detected candidate protein complex *CPC* are analyzed (lines 5-10). Furthermore, if the size of the filtered protein complex *FPC* is larger than or equal to 3, then *FPC* is kept (lines 11-13). As a result, the proteins in the filtered protein complex *FPC* have the same functional annotation term, which indicates whether the proteins in the filtered protein complex perform the same function. Finally, the redundant protein complexes in *FPCs* are eliminated (line 15).
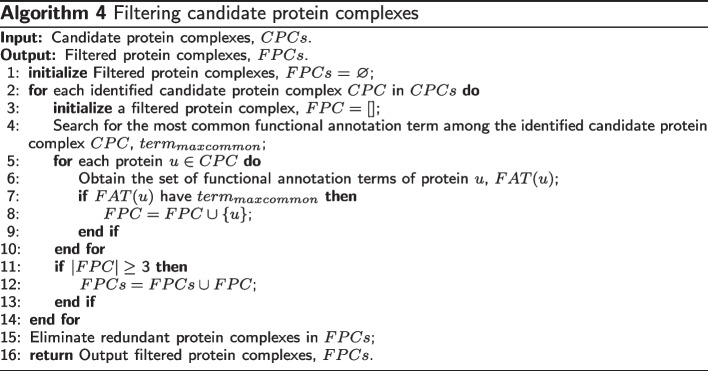


### MP-AHSA algorithm

The MP algorithm has three parameters: the gene co-expression threshold (*GCE*), the inflating of the MCL algorithm (*inflate*), and the ratio of seed nodes (*ratio*), which is used in Algorithm 2. In this paper, we design the adaptive harmony search algorithm (AHSA) to obtains appropriate parameter settings for the MP algorithm.

The harmony search algorithm (HSA) [59] is a new intelligent optimization algorithm. It repeatedly adjusts the solution variables in the harmony memory and converges the objective function with increasing iterations. Compared with other intelligent optimization algorithms, it has the following characteristics:

- It solves variables by harmony simulation without complex coding operations;
- In the HSA, the harmony population is small, which leads to fast operation speed and consumes less memory;
- The convergence and search speed of HSA do rely on have little relation with the initial state of the population, and the result is not affected by the initial state;
- Every harmony in the harmony library participates in variation, and a new harmony is generated by fully using the information in the harmony library.

Traditional HSA mainly have three parameters: *HMCR*, *PAR*, and *FW*. These parameters are usually set as constants, but this suffers from slow convergence speed and low search accuracy. Therefore, we propose an adaptive harmony search algorithm (AHSA) to address this issue.

The improvements to the traditional harmony algorithm have two aspects. The main parameters of the AHSA algorithm are first introduced in Table 2. Meanwhile, according to the definition of these parameters in Table 3, $HMCR_{i}$: The probability of taking a harmony from an existing harmony library, and it controls the global search capability. When the algorithm starts searching, the value of $HMCR_{i}$ is relatively small, and the parameter solution space is searched globally to obtain a better solution. As the number of iterations increases, the value of $HMCR_{i}$ gradually increases to reduce the possibility of a global search. It increases the local search’s possibility and makes the algorithm converge as quickly as possible. $PAR_{i}$: The probability of fine-tuning the harmony obtained from the harmony library controls the probability of local search. If it is not set appropriately, it will affect the convergence speed of the algorithm. Note that when harmony reaches the neighborhood of the optimal solution with the increase of the number of iterations, $PAR_{i}$ should be fine-tuned with a significant probability. When the fitness in the harmony memory is relatively close, $PAR_{i}$ should be significant. $FW_{i}$ is the amplitude of pitch fine-tuning, corresponding to the harmony algorithm’s search step. The harmony vector is scattered in the solution space in the initial stage. A large adaptive is conducive to the global search of the algorithm, and the fitness variance of each harmony in the memory is slight, small adaptive step size is conducive to the local search of the algorithm. For the fine-tuning step size problem, this paper uses the number of iterations and the fitness of the current harmony to adjust the parameter $FW_{i}$. It can be ensured that the AHSA algorithm has strong adaptability and robustness. Second, a novel parameter adaptive adjustment strategy based on $OF_{fitness}$ and the iteration times *i* are designed to improve the searchability and robustness.

**Table 2:** Main parameters of AHSA

ID	Parameters	Abbreviation	Parameter value range and setting
1	Harmony memory	*HMs*	30
2	Harmony memory considering rate	*HMCR*	$HMCR_{max}= 0.95$,$HMCR_{min} = 0.7$
3	Pitch adjusting rate	*PAR*	$PAR_{max}= 0.5,PAR_{min} = 0.1$
4	Fret width	*FW*	$FW_{max} = 0.1,FW_{min} = 0.01$
5	The maximum number of iterations	*Maxiter*	300
6	Gene co-expression threshold	*GCE*	$GCE_{min} = 0.6,GCE_{max} = 0.9$
7	The inflate of MCL	*Inflate*	$inflate_{min} = 0.5,inflate_{max} = 4.0$
8	The ratio of initial seeds	*Ratio*	$ratio_{min} = 0.5,ratio_{max} = 0.9$

**Table 3:** Main modifications of AHSA

Parameters	Adaptive adjustment
$HMCR_{i}$	$HMCR_{i} = HMCR_{min}+i/Maxiter*(HMCR_{max}-HMCR_{min})$
$PAR_{i}$	$PAR_{i} = PAR_{max}-i/Maxiter*(PAR_{max}-PAR_{min})$
$FW_{i}$	$FW_{i} =FW_{min} + (FW_{max} - FW_{min}) * ((fitnessmax - currentfitness)*$
	$$(Maxiter - i) / ((fitnessmax - fitnessmin)*Maxiter))$$

*i* is the times of iteration

Finally, an optimized objective function is defined to guide the AHSA algorithm in searching for the best parameter value for the MP algorithm. We defined the sum of the fitness function of the detected protein complexes is defined as the objective function, as shown in Eq. (10):

10 $$\begin{aligned} OF_{fitness}=\sum _{i=1}^{K} fitness(C_{i}), \end{aligned}$$

where *K* is the number of identified protein complexes, and $fitness(C_{i})$ represents the fitness function of the *i*th identified protein complex ($C_{i}$). The higher the $OF_{fitness}$ of the identified protein complexes, the better the quality. Therefore, the parameter optimization problem of the protein complex detection algorithm is transformed into a problem of finding the set of identified protein complexes with maximum $OF_{fitness}$ within PPI networks.

As a result, the main parameters of the MP algorithm include *GCE*, *inflate*, and *ratio*. In this paper, the AHSA algorithm is used to optimize the MP algorithm’s these parameters (hereafter referred to as the MP-AHSA algorithm). The overall MP-AHSA algorithm is described in Algorithm 5. First, the basic parameters of the AHSA algorithm are set, shown in Table 3 (line 2). Then, the harmony memory *HMs* is initialized based on different parameters and their value ranges, as shown in Table 2 (line 3). Next, the best parameter settings of the MP algorithm are searched repeatedly by creating a new harmony or transforming a harmony from the generated harmony memory (*HMs*) based on $OF_{fitness}$ (lines 4-39). Here, two stages are involved. One selects harmony, and the other adjusts the parameters of the harmony based on the width of the fret *FW*. Additional file 14 shows the flow of the MP-AHSA algorithm to describe it.

In the stage of harmony selection, a variable *R*1 is randomly generated within [0,1], and it is compared with $HMCR_{i}$ based on Table 3 (lines 7-8). If $R1<HMCR_{i}$, a harmony *HM* is selected from the harmony memory (*HMs*) using the roulette wheel selection strategy (line 10). Otherwise, a new harmony is randomly generated according to the parameters and their value ranges in Table 2 (lines 11-17). Then, the maximum and minimum values of $OF_{fitness}$ in *HMs* are determined and recorded as *fitnessmax* and *fitnessmin*, respectively. Next, the value of $FW_{i}$ is calculated based on the number of iterations *i*, *fitnessmax*, and *fitnessmin*. If the harmony is obtained from the harmony memory (lines 10 and 20), a random number *R*2 between [0,1] is generated. The value of $PAR_{i}$ is calculated (lines 21-22). If $R2<PAR$, according to the fine-tuning bandwidth $FW_{i}$, the parameters of the harmony *HM* are adjusted to obtain a new harmony (lines 23-28).

If $R1\le HMCR_{i}$, minor modifications are made to the parameters of the randomly generated harmony $HM_{new}$ based on the fine-tuning bandwidth $FW_{i}$ (lines 29-34). Then, the $OF_{fitness}(HM_{new})$ is calculated according to Eq. (8) (line 35). Suppose the $OF_{fitness}(HM_{new})$ of the newly improvised harmony is better than the $OF_{fitness}(HMs_{min})$ of the worst harmony in *HMs*. In that case, it is replaced to update the harmony memory *HMs* (line 36). Step 3 is repeated many times until a certain termination time *Maxiter* is satisfied (lines 6-39).

Finally, in step 4, according to the $OF_{fitness}$ in Eq. (10), the highest fitness harmony in the harmony memory *HMs* is obtained. It is considered the best clustering output ($HMs_{best}$), and its parameters are appropriate for the input PPI network of the MP algorithm. At this time, this harmony is the identified protein complexes (*IPCs*)(lines 40-42).
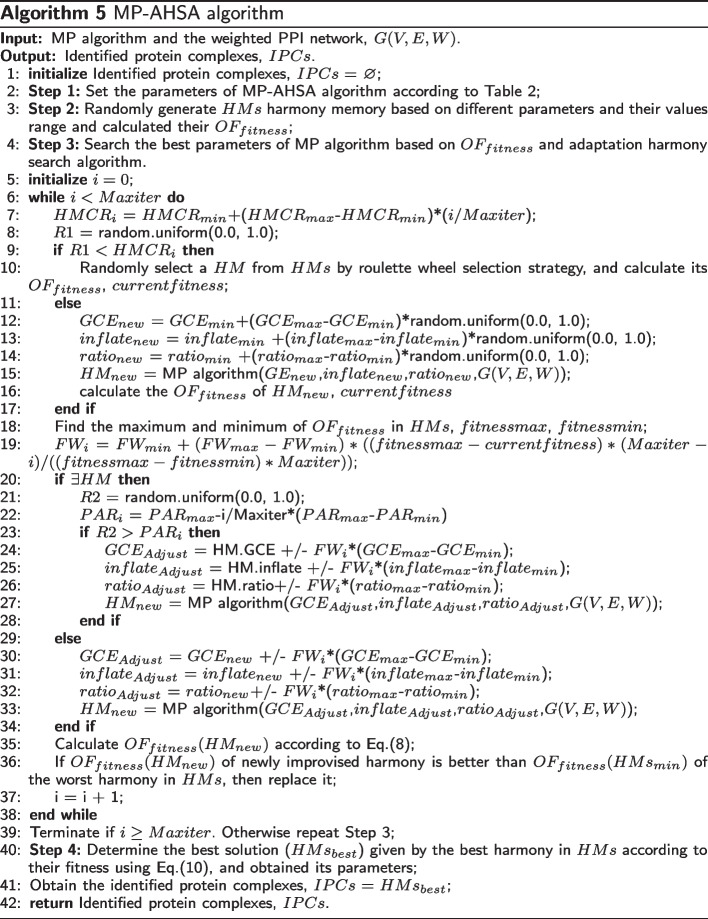


## Results

### Datasets

In this study, three PPI networks are used to conduct the verification experiments: the Collins [60], the Gavin [43], the Krogan [61], String(Saccharomyces cerevisiae, and interaction score $\ge 997$. It can be downloaded from https://cn.string-db.org/cgi/download?sessionId=bjRXzv9e247w), DIP(yeast, and the release date 2015/07/01) [62], and Biogrid (Saccharomyces cerevisiae and these interactions are obtained using different methods from 2020 to 2022 years)) [63] datasets. The detailed properties of these PPI datasets are shown in Table 4. Here, the self-interactions and duplicate interactions are eliminated. If you want to obtain these datasets, please see the Additional files 1, 2, 3, 4, 5, 6 in Supplementary Information.

**Table 4:** Detailed properties of the experimental PPI networks used in the study

Dataset	Nodes	Edges	Density
Collins	1622	9074	0.006902317076
Gavin	1855	7669	0.004459796985
Krogan	2674	7075	0.001979684934
String	1366	5071	0.005439265468
DIP	4696	21822	0.001979524413
Biogrid	4093	13178	0.001573628198

We used two standard protein complexes of the yeast Saccharomyces cerevisiae (SGD) taken from the literature [36]. The properties of these known protein complexes are shown in Table 5. Standard protein complexes 1 consists of the known protein complexes from MIPS [64], SGD [65], TAP06 [43], ALOY [66], CYC2008 [16], and NEWMIPS [67]. Standard protein complexes 2 is also a combined protein complex dataset [68], and it consists of the Wodak database, PINdb and GO complexes [68]. If you want to obtain the two standard protein complexes, please see the Additional files 10, 11 in Supplementary Information.

**Table 5:** Properties of the standard protein complexes used in the study

Datasets	Num	PC	AS
standard protein complexes 1	812	2773	8.92
standard protein complexes 2	1045	2778	8.97

AS: average size of the protein complexes; Num: number of protein complexes; PC: number of proteins

In this study, GO-slim data (available at https://downlo ads.yeastgenome.org) are used to describe the functional similarity of the interactions. Gene expression data is obtained from https://www.ncbi.nlm.nih.gov/sites/GDSbrowser. In addition, subcellular localization data is obtained from https://compartments.jensenlab.org/Dow nloads. If you want to obtain the these biological data, please see the Additional files 7, 8, 9 in Supplementary Information. The stand-alone code of the MP-AHSA algorithm and the datasets are available at: https://github.com/RongquanWang/MP-AHSA.

### Evaluation metrics

In the present study, F-measure, accuracy (ACC), maximum matching ratio (MMR), Jaccard, and total score are used as the computational evaluation metrics to evaluate the performance of protein complex detection algorithms, with *S* and *D* denoting the known and identified protein complexes by a detection method, respectively.

#### Neighborhood affinity

$S_{i}$ represents a standard protein complex in *S*, and $D_{j}$ is a discovered protein complex *D*. Thus, their neighborhood affinity score ($NA(S_{i}, D_{j})$) [69] describes the similarity of two protein complexes $S_{i}$ and $D_{j}$ as defined by Eq. (11):

11 $$\begin{aligned} NA(S_{i},D_{j}) = \frac{|S_{i}\cap D_{j}|^{2}}{|S_{i}|\times |D_{j}|}, \end{aligned}$$

Generally, if $NA(S_{i},D_{j})$ is larger than or equal to 0.2, the protein complexes $S_{i}$ and $D_{j}$ are regarded as matching [6].

#### F-measure

With $N_{sm}$ representing the number of standard protein complexes that match at least one detected protein complex, that is, $N_{sm}=|\{s|s\in S,\exists d\in D,NA(s,d)\ge \omega \}|$, and with $N_{im}$ being the number of detected protein complexes that match at least one standard protein complex, that is, $N_{im}=|\{d|d\in D,\exists s\in S,NA(d,s)\ge \omega \}|$, where $\omega$ is a pre-defined threshold and is usually set as 0.20; then, recall and precision are defined as $recall = \frac{N_{sm}}{|S|}$ and $precision = \frac{N_{im}}{|D|}$, respectively. Finally, the F-measure is represented by the compromise between precision and recall, as defined by Eq. (12):

12 $$\begin{aligned} F-measure=\frac{2\times precision\times recall}{precision + recall}. \end{aligned}$$

#### ACC

$T_{ij}$ is the number of proteins. These proteins are included in the standard protein complex $S_{i}$ and the detected protein complex $D_{j}$. Then, Sn and PPV are calculated by $Sn=\frac{\sum _{i=1}^{|S|} \max _{j=1}^{|I|}\left\{ T_{ij}\right\} }{\sum _{i=1}^{|S|} N_{i}}$ and $PPV=\frac{\sum _{j=1}^{|D|} \max _{i=1}^{|S|}\left\{ T_{ij}\right\} }{\sum _{j=1}^{|D|} \sum _{i=1}^{|S|} T_{ij}}$, respectively. As a result, ACC is defined by Eq. (13):

13 $$\begin{aligned} \begin{array}{l} {ACC=\sqrt{Sn \times PPV}}. \end{array} \end{aligned}$$

#### MMR

The third metric is the MMR [19], which is based on a maximal one-to-one mapping between standard and detected protein complexes. First, each standard protein complex $S_{i}\in S$ and detected protein complex $D_{j}\in D$ are connected by the weight $NA(S_{i}, D_{j})$ edge. The MMR is represented as the sum of the weight of all selected edges divided by |*S*|, as denoted by Eq. (14):

14 $$\begin{aligned} MMR=\frac{\sum _{i=1}^{|S|} \max \limits _{j}NA(S_{i},D_{j})}{|S|}. \end{aligned}$$

#### Fraction

The fraction criterion [19] is an indicator for identification coverage, which measures the percentage of standard protein complexes matched by detected protein complexes. With *S* representing the set of standard protein complexes and *D* being the set of identified protein complexes, the fraction is defined by Eq. (15):

15 $$\begin{aligned} \begin{array}{l} N_{s}=|{s|s\in S,\exists d\in D,NA(d,s)\ge w}|,\\ Frac=\frac{N_{s}}{|S|}. \end{array} \end{aligned}$$

The fraction of gold standard complexes matches at least one detected protein complex. The threshold $\omega$ is set to 0.25, which guarantees that at least half of proteins in a matched standard protein complex are distinguished by at least half of the proteins in a matched detected protein complex.

#### Jaccard

Jaccard is the final category for measuring the clustering methods. Herein, the Jaccard of a standard protein complex $S_{i}\in S$ and a discovered protein complex $D_{j}\in D$ was defined as $Jac(S_{i},D_{j})=\frac{|S_{i}\cap D_{j}|}{|S_{i}\cup D_{j}|}$. For a discovered protein complex $D_{j}$, its Jaccard is $Jac(D_{j}) = max_{S_{i}\in S} Jac(D_{i},S_{i})$, and for a standard protein complex $S_{i}$, its Jaccard is $Jac(S_{i}) = max_{D_{j}\in D} Jac(S_{i},D_{j})$. Then, for detected protein complexes *D*, its average of the weighted Jaccard is $JaccardD=\frac{\sum _{D_{j}\in D} |D_{j}|Jac(D_{j})}{\sum _{D_{j}\in D}|D_{j}|}$. Similarly, for the standard protein complexes *S*, its JaccardS is defined by $JaccardS=\frac{\sum _{S_{i}\in S} |S_{i}|Jac(S_{i})}{\sum _{S_{i}\in S}|S_{i}|}$. Finally, the Jaccard is calculated by Eq. (16):

16 $$\begin{aligned} Jaccard=\frac{2\times (JaccardD\times JaccardS)}{JaccardD + JaccardS}. \end{aligned}$$

#### Total score

To simultaneously consider F-measure, ACC, MMR, Frac, and Jaccard, we use the comprehensive score (total score), given by Eq. (17), to measure the performance of various methods [36].

17 $$\begin{aligned} total\; score=F-measure+ACC+MMR+Frac+Jaccard. \end{aligned}$$

### Comparison with competing methods

To demonstrate the performance of MP-AHSA, we compared it with 14 state-of-the-art protein complex identification methods using the Collins [60], Gavin [43], Krogan core [61] String, DIP [62], and Biogrid [63] datasets. The competing methods used were MCL [11], IPCA [8], COACH [16], CMC [15], ClusterONE [19], PEWCC [23], WPNCA [17], WEC [27], ClusterEPs [37], ClusterSS [38], SE-DMTG [20], MPC-C [36] and GCC-v [25]. Generally, it has been found that the author-suggested parameter settings generate the best results. The values of the parameters used in the different methods are shown in Table 6.

**Table 6:** Parameters of each method used in the study

ID	Year	Algorithm	Parameters
1	2004	MCL	inflation=2
2	2008	IPCA	S=3,P=2,$T_{in}=0.6$
3	2008	COACH	w=0.225
4	2009	CMC	$min\_deg\_ratio$=1,$min\_size$=3, $overlap\_thres$=0.5,$merge_thres$=0.25
5	2010	SPICi	Graph mode=0,minimum support threshold= 0.5, minimum cluster size= 3, minimum density threshold=0.5
6	2012	ClusterONE	Density=auto,Overlap threshold=0.8
7	2013	PEWCC	Overlap=0.8,-r=0.1,Re-join=0.3
8	2015	WPNCA	lambda=0.3,minimum cluster size=3
9	2016	WEC	Balance factor ($\lambda$)=0.8,Edge weight ($T_{w}$)=0.7,Enrichment($T_{e}$)=0.8, Filtering($T_{f}$)=0.9
10	2018	ClusterEPs	NEPs of Complexes(minimum support threshold=0.4,maximum support threshold=0.05); NEPs of non-complexes(maximum support threshold=0.05, minimum support threshold=0.4) ;maximum overlap=0.9,Maximum size of clusters=100
11	2018	ClusterSS	numEpochs = 500,learnRate =0.2,thresholdIn=1.0,thresholdOut=1.02, negativeTime=20, minimum cluster size=3
12	2019	SE-DMTG	minimum cluster size=3
13	2020	MPC-C	Overlap threshold=0.8,minimum cluster size=3
14	2021	GCC-v	Minimum cluster size=3

**Fig. 3:**
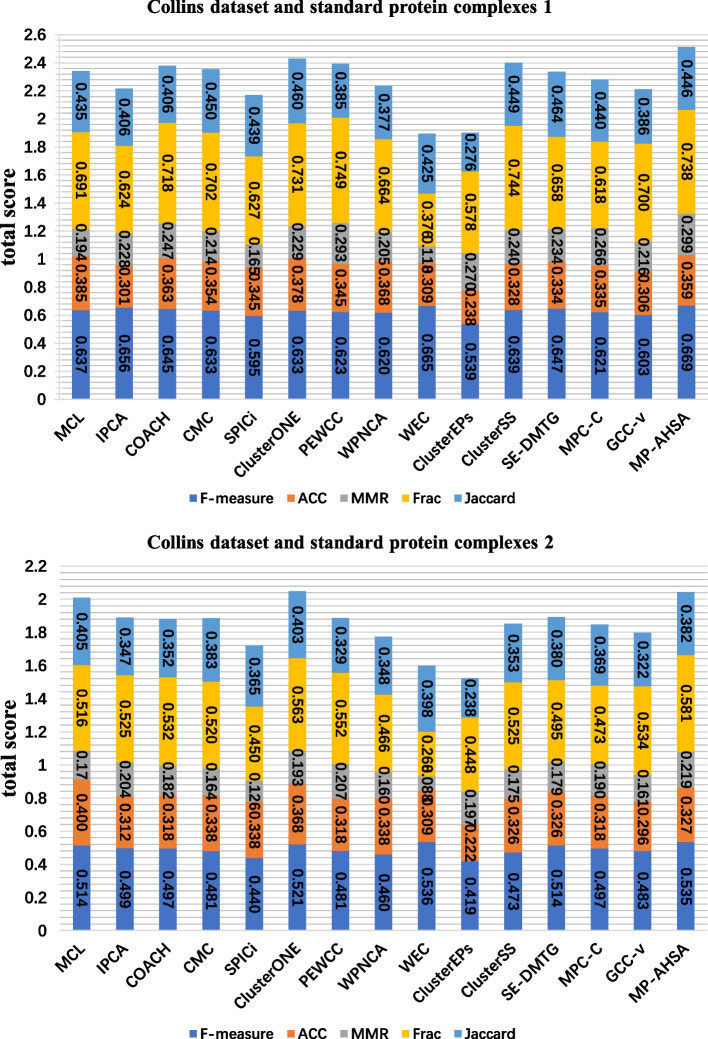
Comparative analysis of identified protein complexes from different approaches in Collins PPI network and two standard protein complexes. The comparative analyses are based on a total score that is a sum of ACC, F-measure, MMR, Frac, and Jaccard (see Evaluation metrics)

**Fig. 4:**
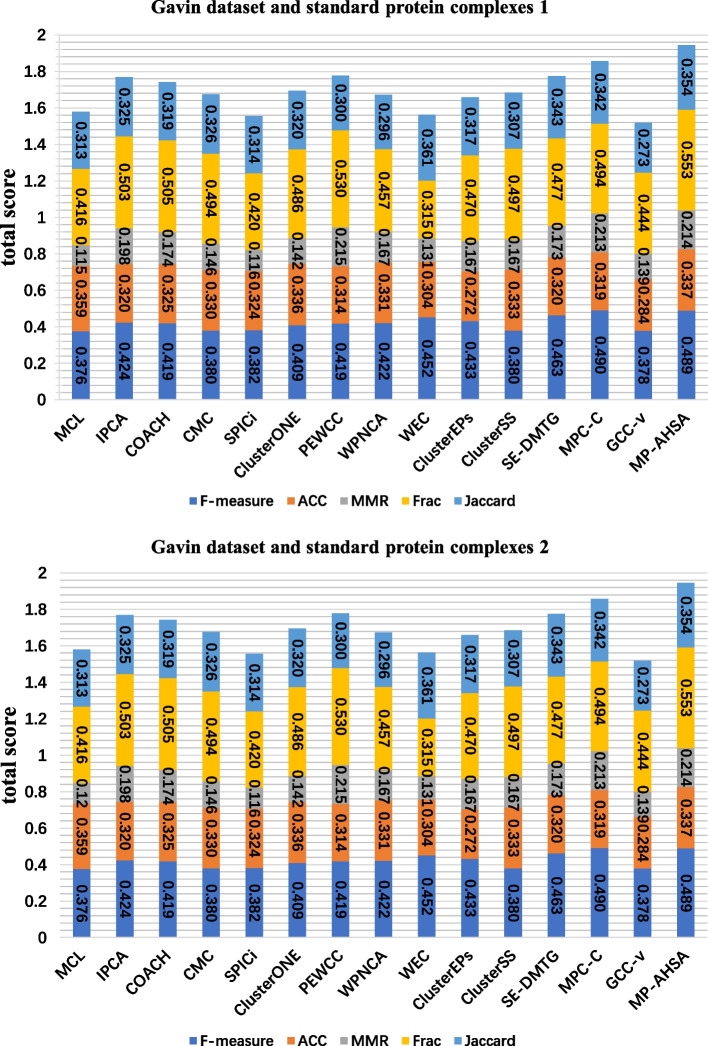
Comparative analysis of identified protein complexes from different approaches in Gavin PPI network and two standard protein complexes. The comparative analyses are based on a total score that is a sum of ACC, F-measure, MMR, Frac, and Jaccard (see Evaluation metrics)

**Fig. 5:**
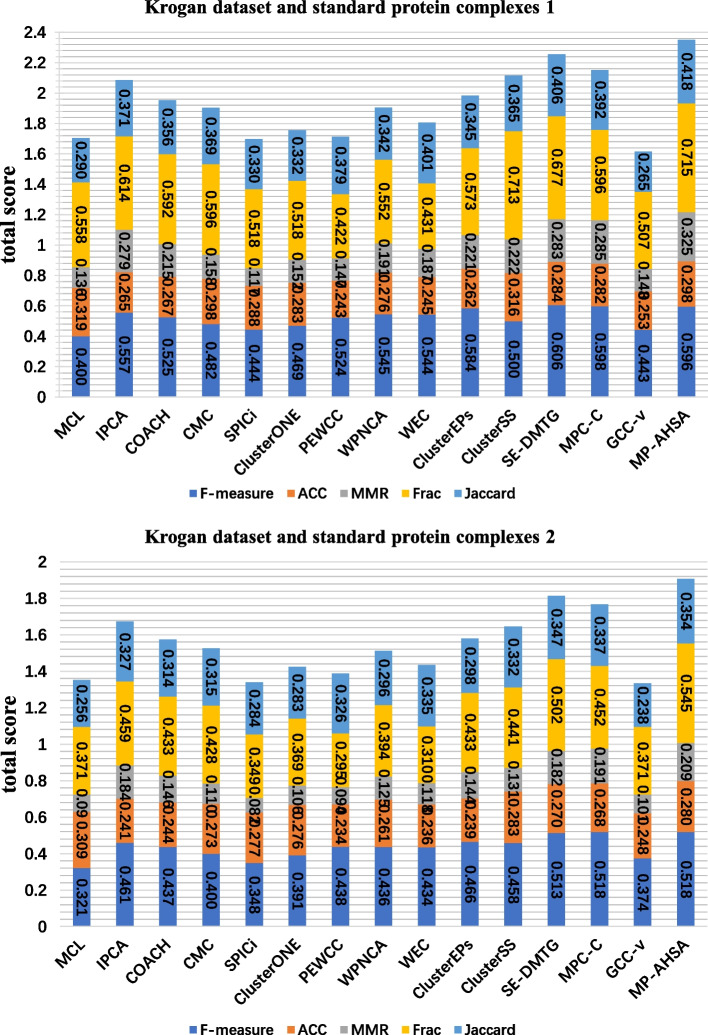
Comparative analysis of identified protein complexes from different approaches in Krogan PPI network and two standard protein complexes. The comparative analyses are based on a total score that is a sum of ACC, F-measure, MMR, Frac, and Jaccard (see Evaluation metrics)

Figures 3, 4 and 5 show the comparison results of 14 competing methods concerning six evaluation metrics (F-measure, ACC, MMR, Frac, Jaccard, and total score). As shown in Figure 3, according to the standard protein complexes 1, MP-AHSA achieves the best results on the F-measure, MMR, and total score statistics. MCL obtains the highest ACC in all PPI datasets. In contrast, MP-AHSA ranks fifth concerning ACC on the Collins dataset, which is lower than the MCL outcome. Meanwhile, PEWCC achieves the best score Frac, and SE-DMTG achieves the highest Jaccard. In contrast, MP-AHSA ranks second and third in terms of Frac and Jaccard, respectively. Meanwhile, when standard protein complexes 2 is used as known protein complexes, MP-AHSA achieves the best performance for MMR and Frac except for ACC, Jaccard, and total score metrics in the Collins dataset. In the Gavin dataset is shown in Figure 4, using standard protein complexes 1 as real protein complexes, PEWCC has a total score value of 2.4973, ranking first among all methods. However, it identifies 664 protein complexes, far more than the number of protein complexes our MP-AHSA recognizes. Moreover, MP-AHSA ranks second concerning the F-measure metric. SE-DMTG obtains the highest Jaccard. When using the standard protein complexes 2, MP-AHSA ranks second in F-measure, second for MMR, second for Jaccard, and second for the ACC statistic. Noteworthily, it achieves the best results on the Frac and total score statistics. In the Krogan core dataset is shown in Figure 5, MP-AHSA achieves the best results regarding the MMR, Frac, Jaccard, and total score static, ranking third on the F-measure statistic in standard protein complexes 1. Furthermore, in standard protein complexes 2, the MP-AHSA algorithm shows the best performance concerning F-measure, MMR, Frac, Jaccard, and total score. It reaches the third-highest level in terms of ACC metrics.

To further verify the performance of our algorithm, we also use three new PPI networks to evaluate these identification algorithms. The evaluation results are shown in Additional files 15, 16, and 17. From the experimental results, we can see that the performance of our algorithm on these datasets is consistent with the performance of the Collins, Gavin, and Krogan datasets. These experimental results show that the MP-AHSA algorithm has strong adaptability and stability to different PPI networks from different datasets.

Altogether, these comparative experimental results show that the MP-AHSA can achieve a higher total score than all the compared methods in most datasets. According to the above-described analysis, multiple PPI datasets and standard protein complexes are used. The MP-AHSA algorithm consistently achieves superior results in most evaluation metrics.

### Case study

**Fig. 6:**
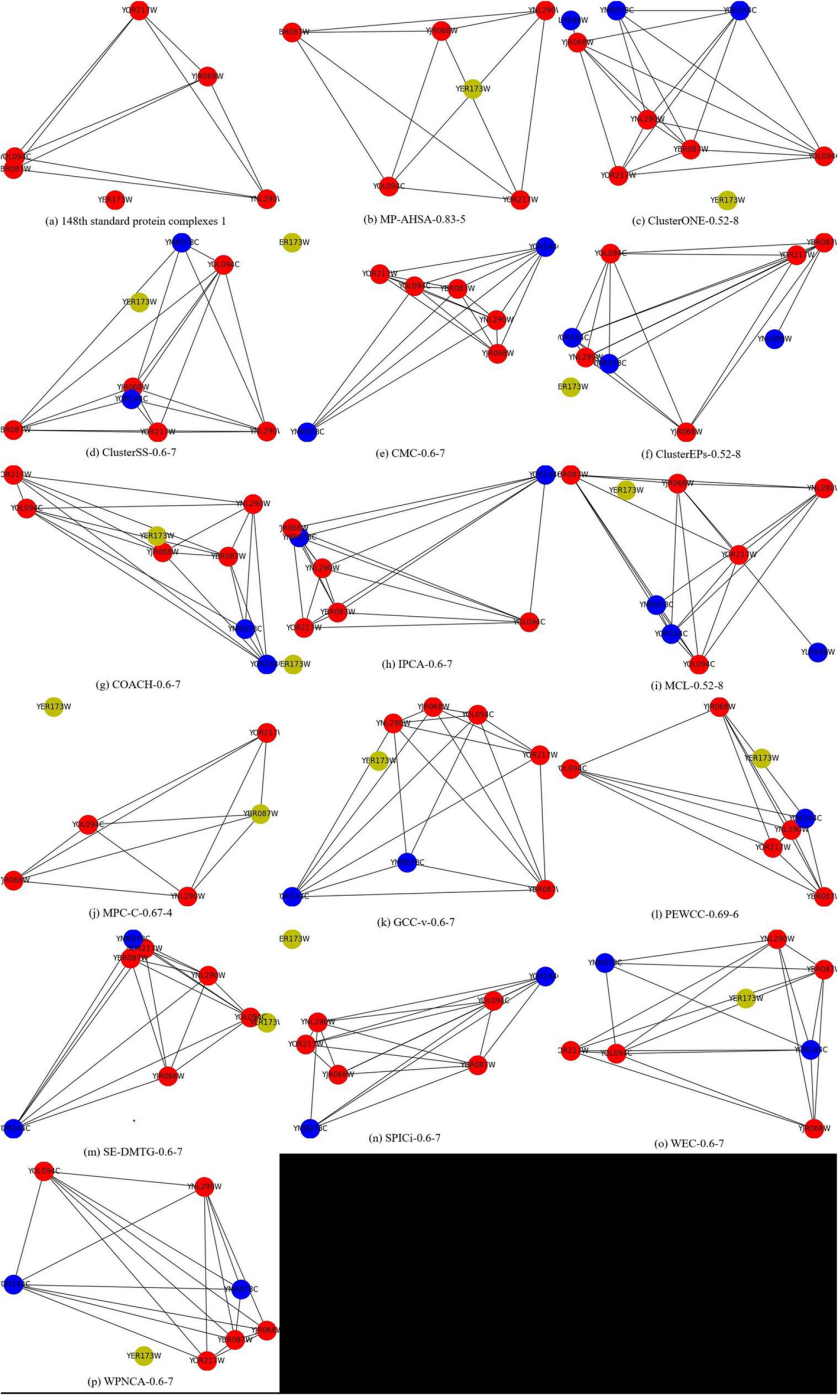
The 390th protein complex in standard protein complexes 1 detected by different methods based on the Gavin dataset. True positive, false-positive, and false-negative proteins are shown in red, blue, and yellow, respectively

In this study, we provide an example of the 148th protein complex comprising 6 proteins in standard protein complexes 1 to show the performance of the described approach. Figure 6 shows the results of different methods used for identifying the protein complex in the Gavin dataset. We define an output format to assist the readers in a more straightforward assessment of the information. For example, MP-AHSA-0.83-5 means that the neighborhood affinity Eq. (11) between our algorithm and the 148th protein complex is 0.83 and that our algorithm contains 5 proteins.

As shown in Figure 6, our method achieves the highest ratio of proteins in the 148th protein complex. Specifically, only MP-AHSA covers the 5 standard proteins and misses one standard protein. ClusterONE, ClusterSS, ClusterEPs, COACH, IPCA, MCL, MPC-C, GCC-v, PEWCC, SE-DMTG, WEC, and WPNCA all miss a standard protein. Moreover, ClusterONE, ClusterSS, CMC, ClusterEPs, COACH, IPCA, MCL, GCC-v, PEWCC, SE-DMTG, SPICi, WEC, and WPNCA only covered part of the standard proteins and detected some false-positive proteins. In conclusion, our algorithm only misses a standard protein to the 148th standard protein complex and shows the best predictive performance.

## Discussion

### Functional enrichment analysis

Additionally, we also use the proportion of biologically significant protein complexes to evaluate the detected protein complexes. The p-value of a protein complex *C* with respect to a functional group *F* is denoted by Eq. (18):

18 $$\begin{aligned} p-value=1-\sum _{i=0}^{k-1} \frac{\left( {\begin{array}{c}|F|\\ i\end{array}}\right) \left( {\begin{array}{c}|V|-|F|\\ |C|-i\end{array}}\right) }{\left( {\begin{array}{c}|V|\\ |C|\end{array}}\right) }, \end{aligned}$$

where *k* represents the number of proteins covered in *C* and *F*, and *V* represents the set of proteins in a PPI network. If the smallest p-value of *C* concerning all functional groups is smaller than 0.01, the detected protein complex *C* was regarded as biologically significant. Herein, we use the fast tool LAGO [70] to compute the p-value of the detected protein complexes.

### Comparison with functional enrichment

To further estimate the effectiveness of the MP-AHSA algorithm, we investigate the biological significance of the identified protein complexes. Here, each protein complex is identified by the various methods associated with a p-value for GO annotation. The percentage of biological significant protein complexes detected by different methods is shown in Table 7 and Additional file 18. Herein, the number and percentage of the identified complexes, for which p-value was in the range of $\le$E-20, [E-20, E-15), [E-15, E-10), [E-10, E-5), [E-5, 0.01), $\le$0.01, are listed in Table 7 and Additional file 18.

**Table 7:** Functional enrichment analysis of protein complexes detected by different methods in Collins, Gavin and Krogan datasets

Method	${\mathbf{ < }}\left( {\varvec{E}{\mathbf{ - 20}}} \right)$	[E-20,E-15)	[E-15,E-10)	[E-10,E-5)	[E-5,0.01)	$\leqslant {\mathbf{0.01}}$
Collins dataset						
MCL	62(39.24%)	9(5.7%)	25(15.82%)	40(25.32%)	4(2.53%)	140(88.61%)
IPCA	108(31.58%)	37(10.82%)	63(18.42%)	97(28.36%)	16(4.68%)	321(93.86%)
COACH	64(25.5%)	22(8.76%)	39(15.54%)	80(31.87%)	14(5.58%)	219(87.25%)
CMC	54(30.51%)	17(9.6%)	22(12.43%)	63(35.59%)	8(4.52%)	164(92.66%)
SPICi	62(51.24%)	10(8.26%)	19(15.7%)	25(20.66%)	3(2.48%)	**119(98.35%)**$^{1st}$
ClusterONE	47(23.15%)	19(9.36%)	45(22.17%)	61(30.05%)	11(5.42%)	183(90.15%)
PEWCC	128(30.05%)	21(4.93%)	104(24.41%)	120(28.17%)	18(4.23%)	391(91.78%)
WPNCA	90(33.46%)	33(12.27%)	61(22.68%)	52(19.33%)	7(2.6%)	243(90.33%)
WEC	394(40.74%)	81(8.38%)	174(17.99%)	261(26.99%)	23(2.38%)	933(96.48%)
ClusterEPs	4(0.68%)	13(2.21%)	95(16.18%)	350(59.63%)	74(12.61%)	536(91.31%)
ClusterSS	22(10.05%)	19(8.68%)	48(21.92%)	93(42.47%)	18(8.22%)	200(91.32%)
	28(12.96%)	25(11.57%)	45(20.83%)	85(39.35%)	19(8.8%)	202(93.52%)
SE-DMTG	58(34.73%)	22(13.17%)	29(17.37%)	46(27.54%)	6(3.59%)	161(96.41%)
MPC-C	75(27.37%)	35(12.77%)	49(17.88%)	86(31.39%)	10(3.65%)	255(93.07%)
GCC-v	11(5.16%)	19(8.92%)	28(13.15%)	107(50.23%)	29(13.62%)	194(91.08%)
MP-AHSA	75(27.17%)	36(13.04%)	48(17.39%)	94(34.06%)	15(5.43%)	268(97.1%)$^{2nd}$
Gavin dataset						
MCL	24(10.91%)	22(10.0%)	35(15.91%)	72(32.73%)	22(10.0%)	175(79.55%)
IPCA	121(26.08%)	58(12.5%)	70(15.09%)	106(22.84%)	41(8.84%)	396(85.34%)
COACH	124(34.35%)	34(9.42%)	52(14.4%)	83(22.99%)	18(4.99%)	311(86.15%)
CMC	71(24.15%)	15(5.1%)	40(13.61%)	76(25.85%)	21(7.14%)	223(75.85%)
SPICi	47(24.87%)	15(7.94%)	30(15.87%)	54(28.57%)	17(8.99%)	163(86.24%)
ClusterONE	52(20.16%)	11(4.26%)	36(13.95%)	78(30.23%)	20(7.75%)	197(76.36%)
PEWCC	76(11.45%)	51(7.68%)	108(16.27%)	224(33.73%)	77(11.6%)	536(80.72%)
WPNCA	128(26.45%)	32(6.61%)	100(20.66%)	158(32.64%)	19(3.93%)	437(90.29%)
WEC	261(28.87%)	82(9.07%)	151(16.7%)	234(25.88%)	66(7.3%)	794(87.83%)
ClusterEPs	74(27.31%)	35(12.92%)	47(17.34%)	62(22.88%)	22(8.12%)	240(88.56%)
ClusterSS	27(6.47%)	24(5.76%)	57(13.67%)	178(42.69%)	50(11.99%)	336(80.58%)
	30(7.59%)	21(5.32%)	68(17.22%)	165(41.77%)	42(10.63%)	326(82.53%)
SE-DMTG	82(35.65%)	35(15.22%)	38(16.52%)	48(20.87%)	13(5.65%)	216(93.91%)$^{2nd}$
MPC-C	124(31.16%)	38(9.55%)	58(14.57%)	152(38.19%)	10(2.51%)	**382(95.98%)**$^{1st}$
GCC-v	13(4.45%)	15(5.14%)	27(9.25%)	101(34.59%)	44(15.07%)	200(68.49%)
MP-AHSA	100(27.17%)	30(8.15%)	58(15.76%)	125(33.97%)	32(8.7%)	345(93.75%)
Krogan dataset						
MCL	31(8.38%)	23(6.22%)	40(10.81%)	118(31.89%)	31(8.38%)	243(65.68%)
IPCA	101(17.35%)	70(12.03%)	90(15.46%)	218(37.46%)	39(6.7%)	518(89.0%)
COACH	68(19.71%)	33(9.57%)	53(15.36%)	118(34.2%)	27(7.83%)	299(86.67%)
CMC	36(13.64%)	19(7.2%)	38(14.39%)	92(34.85%)	21(7.95%)	206(78.03%)
SPICi	10(4.46%)	17(7.59%)	42(18.75%)	68(30.36%)	25(11.16%)	162(72.32%)
ClusterONE	34(14.17%)	16(6.67%)	34(14.17%)	109(45.42%)	14(5.83%)	207(86.25%)
PEWCC	146(37.53%)	50(12.85%)	71(18.25%)	95(24.42%)	16(4.11%)	**378(97.17%)**$^{1st}$
WPNCA	106(28.73%)	52(14.09%)	61(16.53%)	114(30.89%)	17(4.61%)	350(94.85%)
WEC	171(33.14%)	64(12.4%)	88(17.05%)	141(27.33%)	19(3.68%)	483(93.6%)
ClusterEPs	53(12.93%)	32(7.8%)	57(13.9%)	237(57.8%)	14(3.41%)	393(95.85%)
ClusterSS	35(7.73%)	33(7.28%)	50(11.04%)	188(41.5%)	34(7.51%)	340(75.06%)
	42(17.43%)	33(13.69%)	43(17.84%)	92(38.17%)	12(4.98%)	222(92.12%)
SE-DMTG	33(9.14%)	33(9.14%)	69(19.11%)	173(47.92%)	23(6.37%)	331(91.69%)
MPC-C	93(20.39%)	70(15.35%)	110(24.12%)	160(35.09%)	7(1.54%)	440(96.49%)$^{2nd}$
GCC-v	11(3.53%)	9(2.88%)	28(8.97%)	148(47.44%)	29(9.29%)	225(72.12%)
MP-AHSA	75(14.71%)	35(6.86%)	90(17.65%)	232(45.49%)	27(5.29%)	459(90.0%)

The highest score of each row are shown in bold

As Table 7 shows, in the Collins dataset, our MP-AHSA achieves second in the percentage of biologically significant protein complexes, reaching 97.1$\%$, which is lower than that of the SPICi method. However, SPICi only detected 121 protein complexes, which is also why it can get a higher percentage of biologically significant protein complexes than the output of MP-AHSA. In the Gavin dataset, MPC-C achieves the best percentage of biologically significant protein complexes, which is better than MP-AHSA based on Table 7. In the Krogan dataset, PEWCC achieves the best percentage of biologically significant protein complexes. It outperforms our MP-AHSA algorithm. Two reasons are: (1) MP-AHSA predicted more detected protein complexes than PEWCC, and (2) the average size of the detected protein complexes identified by PEWCC is more significant than that of MP-AHSA. In particular, the average size of the detected protein complexes predicted by PEWCC and MP-AHSA is 10.28 and 6.6, respectively. In contrast, the average size of standard protein complexes is minimal [20]. Note that as the p-value of an identified protein complex is closely associated with the size, the p-value gradually decreases as the size of the detected protein complexes increases [16, 17, 20].

Meanwhile, we also calculate the p-value of three new PPI networks to obtain functional enrichment analysis to measure the biologically significant of identified protein complexes by different algorithms. The evaluation results are shown in Additional file 18. From the experimental results, we can see that the performance of our algorithm on these datasets is the best in all protein complex detection methods. These experimental results illustrate that the MP-AHSA algorithm can identify biological protein complexes, and our method has strong stability in different PPI networks.

In conclusion, MP-AHSA can identify more protein complexes with significant GO terms. Although some of those identified protein complexes are not known, they are more likely to be experimentally verified as factual protein complexes by biologists. Therefore, based on the p-value results, the MP-AHSA algorithm can effectively detect biologically meaningful protein complexes.

## Conclusions

Detection of protein complexes is essential to understanding cellular mechanisms. In this study, the MP-AHSA algorithm is proposed to identify protein complexes. First, a weighted PPI network is designed using the TCSS method based on functional annotations. Then, local protein complex cores are identified based on co-subcellular localization and gene co-expression datas. Global protein complex cores are detected using the MCL method. Second, a new fitness function is defined to guide mining attachment proteins. Third, all candidate protein complexes are filtered to obtain the filtered protein complexes. Finally, the AHSA algorithm is used to determine the parameter settings of the MP algorithm based on the input PPI network. The experimental results on widely used PPI networks indicate that the MP-AHSA algorithm outperforms 14 competing methods and can effectively detect biologically meaningful protein complexes. In the future, advanced machine learning techniques, such as ensemble learning and graph attention networks will be applied to this field.

## Supplementary Information

**Additional file 1.** Collins PPI network.

**Additional file 2.** Gavin PPI network.

**Additional file 3.** Krogan PPI network.

**Additional file 4.** String PPI network.

**Additional file 5.** DIP PPI network.

**Additional file 6.** Biogrid PPI network.

**Additional file 7.** Gene expression data.

**Additional file 8.** Go slim mapping.

**Additional file 9.** Subcellular localization data.

**Additional file 10.** Standardcomplexes1.

**Additional file 11.** Standardcomplexes2

**Additional file 12.** An example diagram to describe the Algorithm 2.

**Additional file 13.** An example diagram to describe the Algorithm 3.

**Additional file 14.** An example diagram to describe the Algorithm 5.

**Additional file 15.** Experimental results on String dataset.

**Additional file 16.** Experimental results on DIP dataset.

**Additional file 17.** Experimental results on Biogrid dataset.

**Additional file 18.** Functional enrichment analysis of protein complexes detected in String, DIP and Biogrid datasets.

## Data Availability

The datasets and the stand-alone code of the MP-AHSA algorithm are available in https://github.com/RongquanWang/MP-AHSA or it is available from the corresponding author on reasonable request.

## Abbreviations

PPI: Protein-protein interaction
MP: Multiple properties
MP-AHSA: Multiple properties with an adaptation harmony search algorithm
MCL: Markov cluster algorithm
TAP-MS: Tandem affinity purification with mass spectrometry
TCSS: Topological clustering semantic similarity
CC: Cellular component
BP: Biological process
MP: Molecular function
SLD: Subcellular localization data
GED: Gene expression data
HSA: Harmony search algorithm
GCE: Gene co-expression threshold
inflate: The inflate of the MCL algorithm
ratio: The ratio of seed nodes
HMs: Harmony memory
HMCR: Harmony memory considering rate
PAR: Pitch adjusting rate
FW: Fret width
Maxiter: The maximum number of iterations
ACC: Accuracy
MMR: Maximum matching ratio
Frac: Fraction
total score: The composite score of F-measure, ACC, MMR, Frac and Jaccard
Num: Number of protein complexes
AS: Average size of the protein complexes
PC: Number of proteins.
