# Correction: Detecting protein complexes with multiple properties by an adaptive harmony search algorithm

**DOI:** 10.1186/s12859-022-05049-3

**Published:** 2022-11-18

**Authors:** Rongquan Wang, Caixia Wang, Huimin Ma

**Affiliations:** 1 School of Computer and Communication Engineering, University of Science and Technology Beijing, No. 30 Xueyuan Road, Haidian District, Beijing, 100083 China; 2 School of International Economics, China Foreign Affairs University, 24 Zhanlanguan Road, Xicheng District, Beijing, 100037 China

## Correction to: BMC Bioinformatics (2022) 23:414 10.1186/s12859-022-04923-4

Following publication of the original article [1], the authors would like to remove the symbol of equal contribution behind the names.

The original article [1] has been corrected.
