# Supplementary figures and images for: Detecting protein complexes with multiple properties by an adaptive harmony search algorithm
Source: BMC Bioinformatics. 2022 Oct 7;23:414. doi: 10.1186/s12859-022-04923-4 (PMC9541083; doi:10.1186/s12859-022-04923-4)

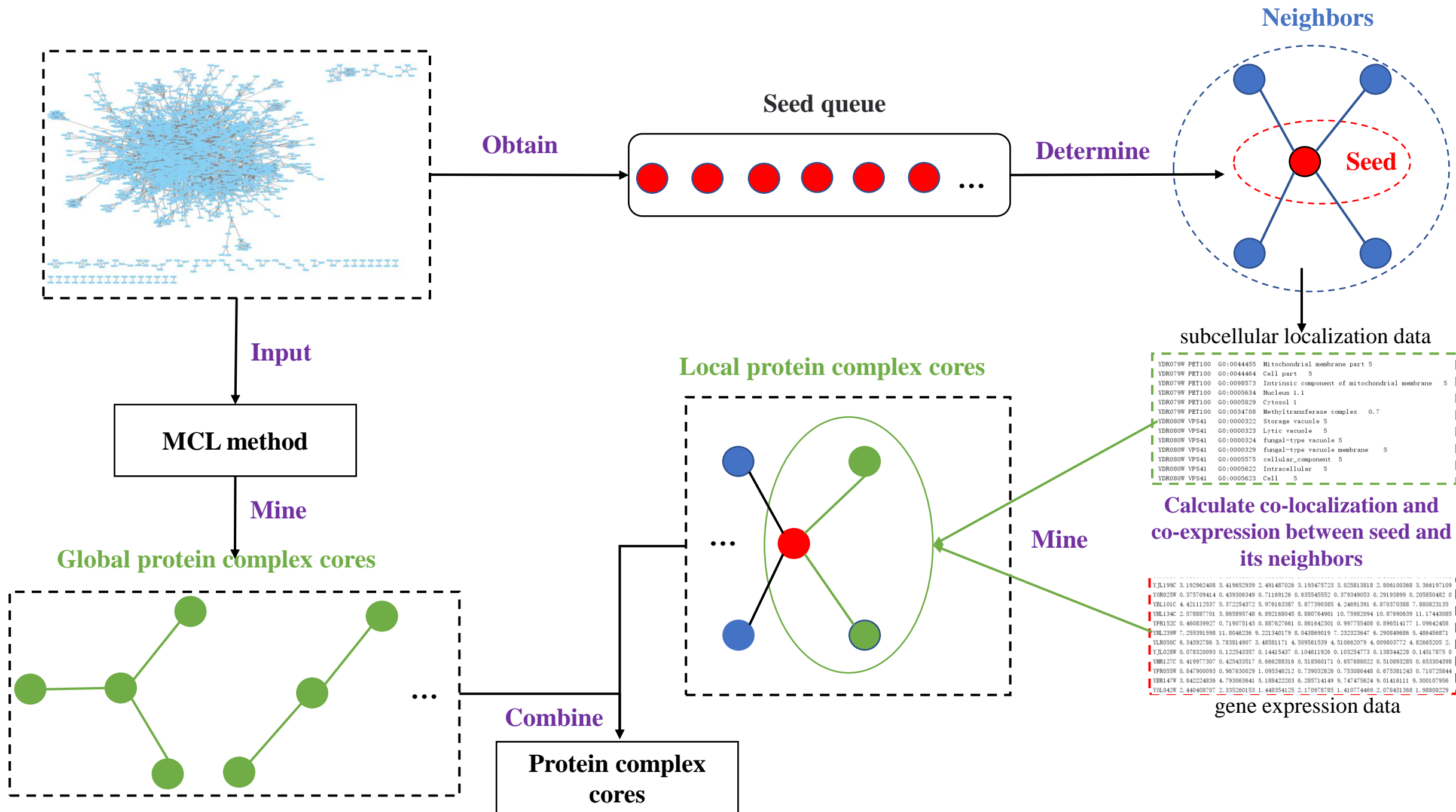

Supplement: Supplementary file 1 — Additional file 1. Collins PPI network. [file 12859_2022_4923_MOESM1_ESM.pdf]

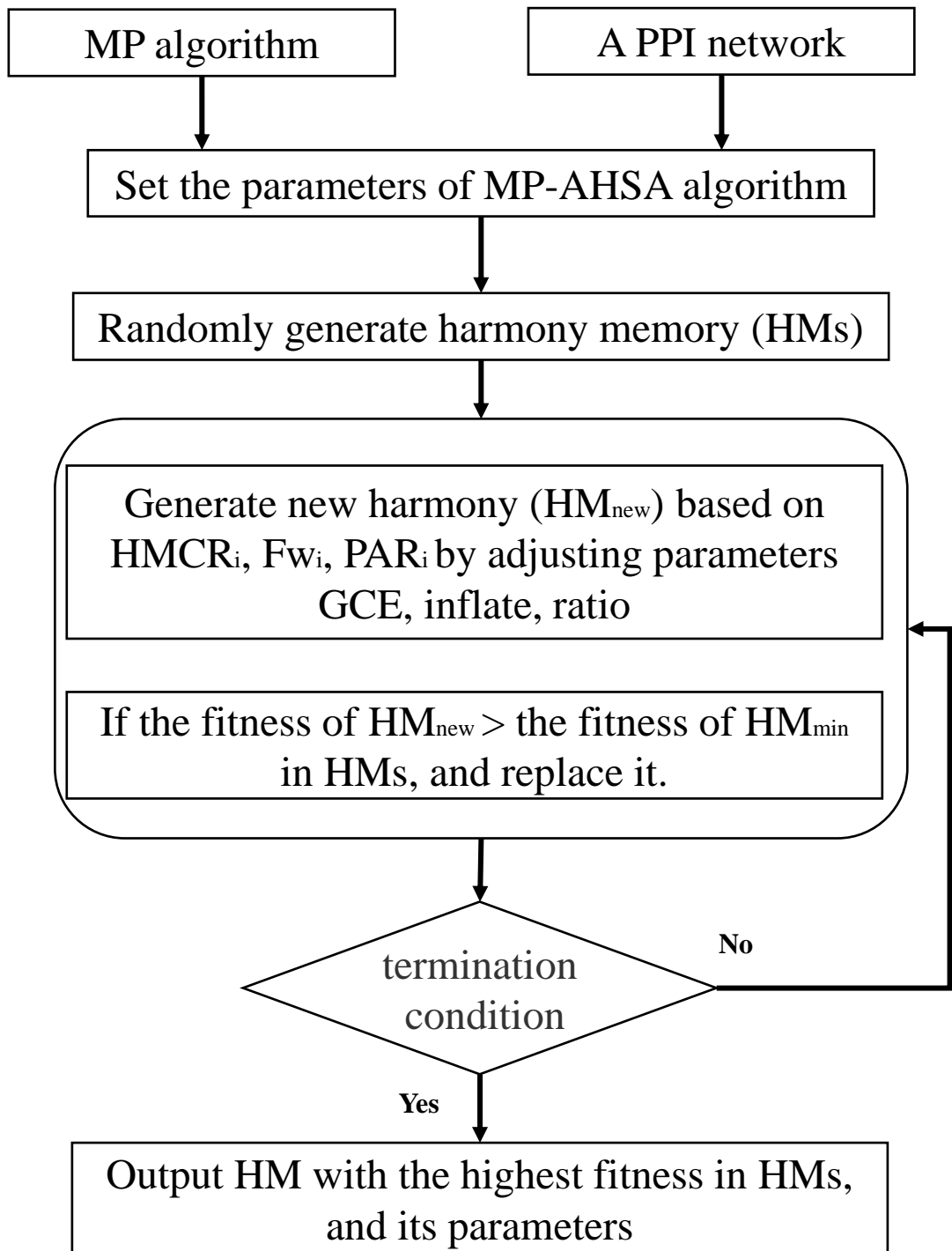

Supplement: Supplementary file 3 — Additional file 3. Krogan PPI network. [file 12859_2022_4923_MOESM3_ESM.pdf]

DIP dataset and standard protein complexes 1

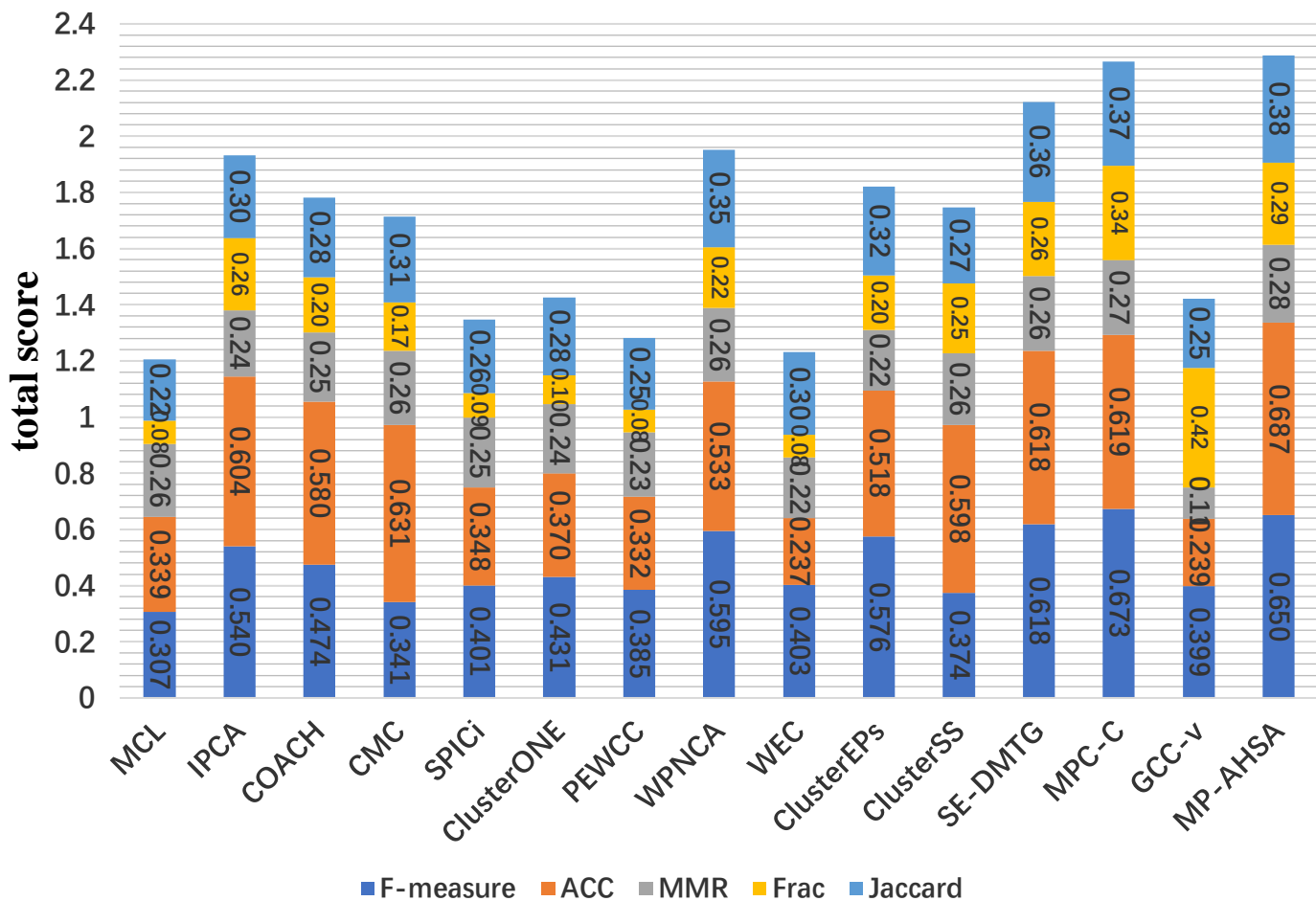

DIP dataset and standard protein complexes 2

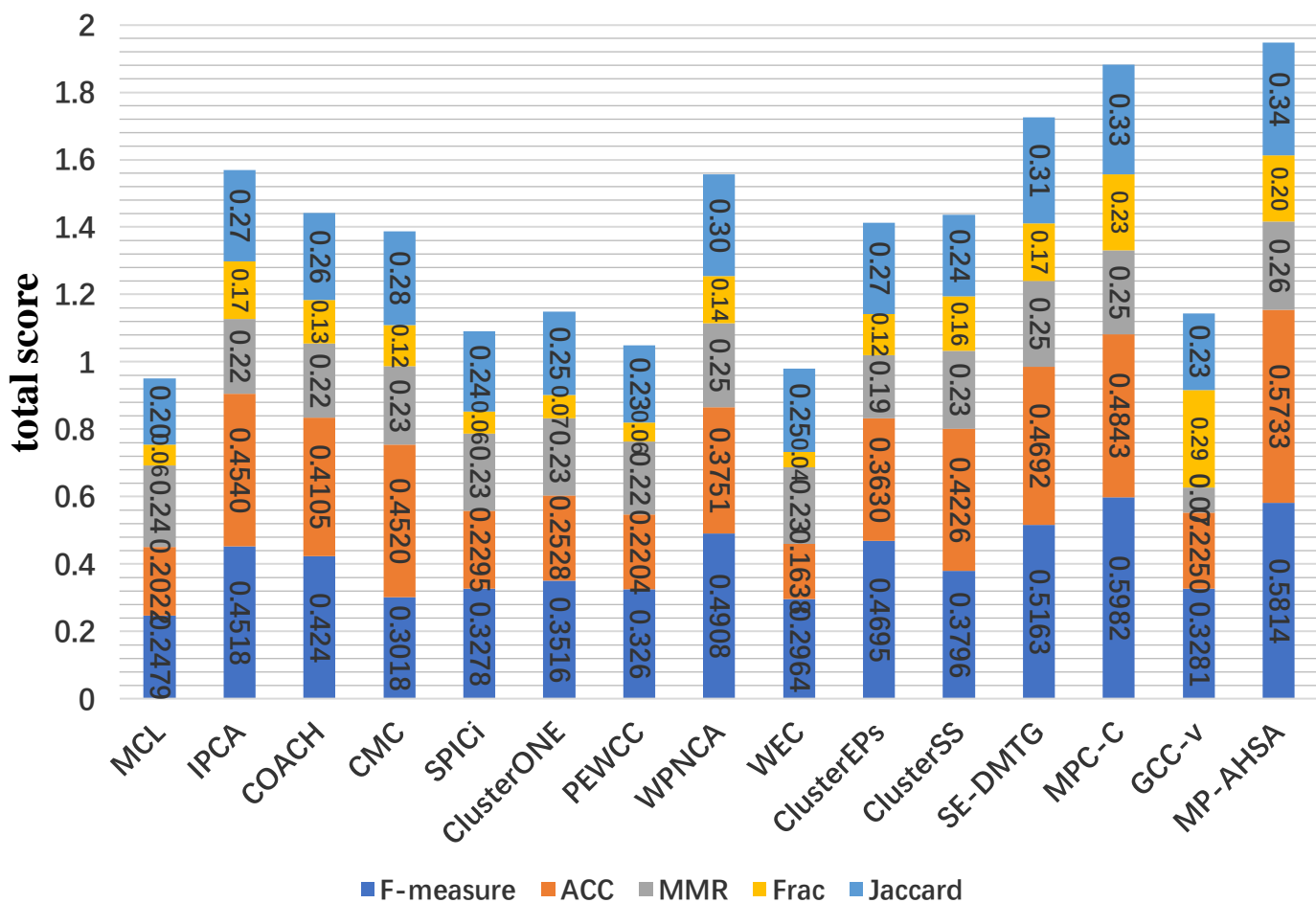

Supplement: Supplementary file 6 — Additional file 6. Biogrid PPI network. [file 12859_2022_4923_MOESM6_ESM.pdf]
