# Supplementary material for: Detecting protein complexes with multiple properties by an adaptive harmony search algorithm
Source: BMC Bioinformatics. 2022 Oct 7;23:414. doi: 10.1186/s12859-022-04923-4 (PMC9541083; doi:10.1186/s12859-022-04923-4)
Supplement: Supplementary file 2 — Additional file 2. Gavin PPI network. [file 12859_2022_4923_MOESM2_ESM.pdf]

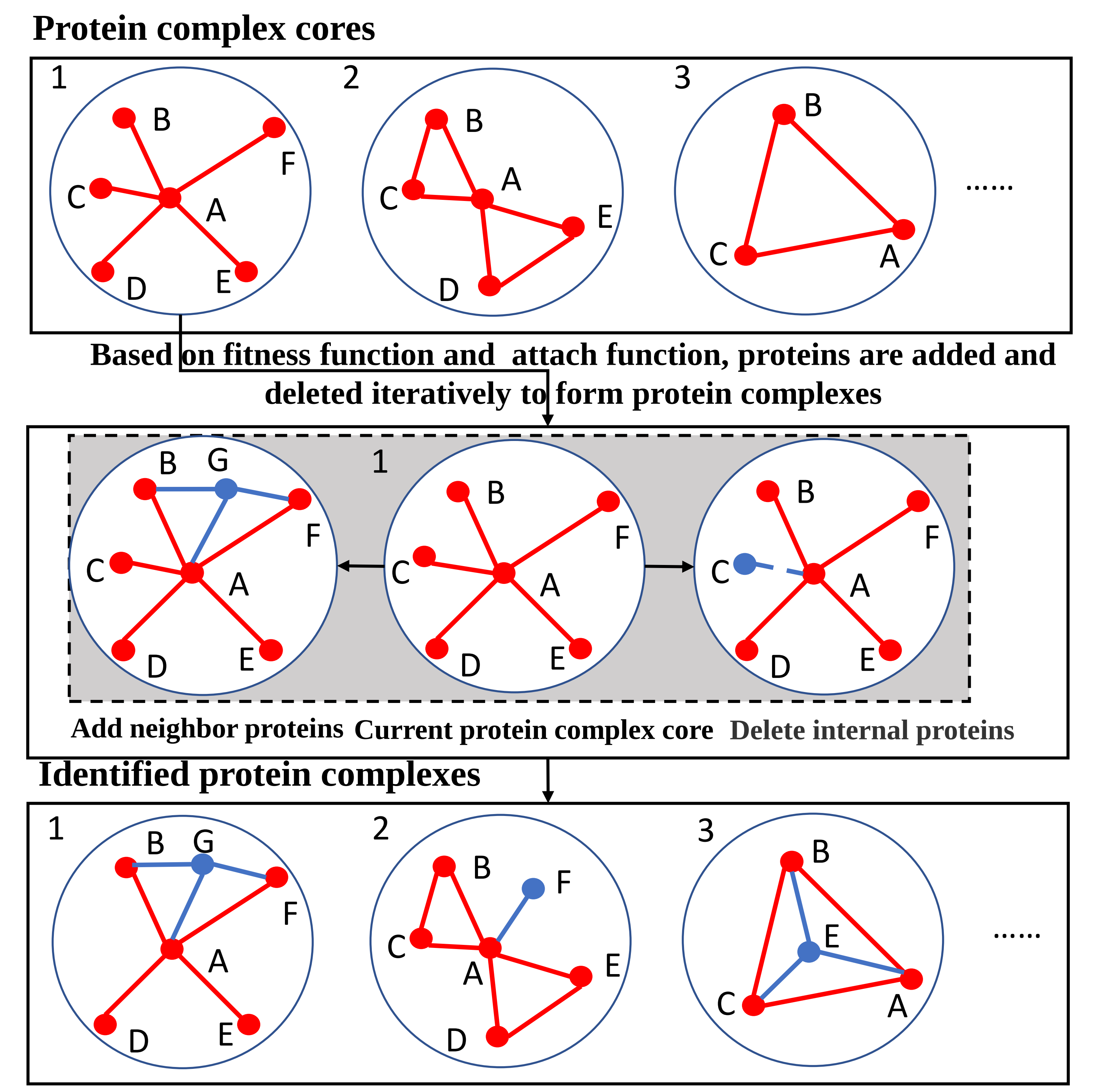
Protein complex cores
1
B
F
C
A
D
E
2
B
A
C
E
D
3
B
C
A
......
Based on fitness function and attach function, proteins are added and deleted iteratively to form protein complexes
B
G
1
F
C
A
D
E
B
F
C
A
D
E
B
F
C
A
D
E
Add neighbor proteins
Current protein complex core
Delete internal proteins
Identified protein complexes
1
B
G
F
A
D
E
2
B
F
C
A
E
D
3
B
E
C
A
......
