# Supplementary material for: Detecting protein complexes with multiple properties by an adaptive harmony search algorithm
Source: BMC Bioinformatics. 2022 Oct 7;23:414. doi: 10.1186/s12859-022-04923-4 (PMC9541083; doi:10.1186/s12859-022-04923-4)
Supplement: Supplementary file 4 — Additional file 4. String PPI network. [file 12859_2022_4923_MOESM4_ESM.pdf]

Table 10 Functional enrichment analysis of protein complexes detected by different methods in different datasets

Method	$<$E-20	[E-20,E-15)	[E-15,E-10)	[E-10,E-5)	[E-5,0.01)	$\leqslant$0.01
String dataset						
MCL	28(19.05%)	16(10.88%)	26(17.69%)	66(44.9%)	3(2.04%)	139(94.56%)
IPCA	64(21.84%)	22(7.51%)	48(16.38%)	96(32.76%)	9(3.07%)	239(81.57%)
COACH	30(15.23%)	19(9.64%)	33(16.75%)	82(41.62%)	1(0.51%)	165(83.76%)
CMC	30(20.83%)	7(4.86%)	15(10.42%)	66(45.83%)	20(13.89%)	138(95.83%)
SPICi	39(34.21%)	11(9.65%)	20(17.54%)	39(34.21%)	1(0.88%)	110(96.49%)
ClusterONE	55(26.57%)	19(9.18%)	35(16.91%)	86(41.55%)	3(1.45%)	198(95.65%)
PEWCC	74(22.09%)	64(19.1%)	67(20.0%)	123(36.72%)	7(2.09%)	335(100.0%)
WPNCA	22(15.17%)	10(6.9%)	27(18.62%)	78(53.79%)	7(4.83%)	144(99.31%)
WEC	61(10.25%)	83(13.95%)	93(15.63%)	157(26.39%)	62(10.42%)	456(76.64%)
ClusterEPs	23(7.72%)	25(8.39%)	89(29.87%)	155(52.01%)	3(1.01%)	295(98.99%)
ClusterSS	44(11.52%)	29(7.59%)	47(12.3%)	111(29.06%)	45(11.78%)	276(72.25%)
	53(27.32%)	17(8.76%)	31(15.98%)	71(36.6%)	7(3.61%)	179(92.27%)
SE-DMTG	37(21.76%)	21(12.35%)	35(20.59%)	70(41.18%)	4(2.35%)	167(98.24%)
MPC-C	54(24.22%)	30(13.45%)	30(13.45%)	61(27.35%)	10(4.48%)	185(82.96%)
GCC-v	6(2.99%)	7(3.48%)	32(15.92%)	128(63.68%)	11(5.47%)	184(91.54%)
MP-AHSA	56(20.82%)	36(13.38%)	52(19.33%)	116(43.12%)	9(3.35%)	**269(100.0%)**^{1st}
DIP dataset						
MCL	40(6.88%)	32(5.51%)	73(12.56%)	210(36.14%)	48(8.26%)	403(69.36%)
IPCA	112(7.47%)	137(9.14%)	223(14.88%)	518(34.56%)	149(9.94%)	1139(75.98%)
COACH	68(7.53%)	68(7.53%)	137(15.17%)	349(38.65%)	92(10.19%)	714(79.07%)
CMC	86(7.85%)	47(4.29%)	96(8.76%)	309(28.19%)	141(12.86%)	679(61.95%)
SPICi	110(27.92%)	48(12.18%)	62(15.74%)	116(29.44%)	25(6.35%)	361(91.62%)
ClusterONE	22(6.23%)	56(15.86%)	51(14.45%)	100(28.33%)	26(7.37%)	255(72.24%)
PEWCC	78(22.1%)	57(16.15%)	57(16.15%)	92(26.06%)	17(4.82%)	301(85.27%)
WPNCA	128(26.45%)	32(6.61%)	100(20.66%)	158(32.64%)	19(3.93%)	437(90.29%)
WEC	51(19.62%)	33(12.69%)	41(15.77%)	63(24.23%)	17(6.54%)	205(78.85%)
ClusterEPs	38(5.33%)	100(14.03%)	176(24.68%)	244(34.22%)	51(7.15%)	609(85.41%)
ClusterSS	115(4.78%)	135(5.62%)	232(9.65%)	669(27.83%)	320(13.31%)	1471(61.19%)
	87(4.04%)	120(5.57%)	304(14.1%)	737(34.18%)	233(10.81%)	1481(68.69%)
SE-DMTG	96(11.75%)	73(8.94%)	132(16.16%)	324(39.66%)	62(7.59%)	687(84.09%)
MPC-C	144(8.77%)	160(9.74%)	343(20.89%)	592(36.05%)	136(8.28%)	1375(83.74%)
GCC-v	9(1.78%)	11(2.17%)	56(11.07%)	209(41.3%)	57(11.26%)	342(67.59%)
MP-AHSA	133(14.94%)	75(8.43%)	135(15.17%)	416(46.74%)	71(7.98%)	**830(93.26%)**^{1st}
Biogrid dataset						
MCL	0(0.0%)	0(0.0%)	0(0.0%)	84(100.0%)	0(0.0%)	84(100.0%)
IPCA	119(4.64%)	104(4.05%)	195(7.6%)	1361(53.04%)	174(6.78%)	1953(76.11%)
COACH	0(0.0%)	0(0.0%)	0(0.0%)	403(100.0%)	0(0.0%)	403(100.0%)
CMC	0(0.0%)	0(0.0%)	0(0.0%)	0(0.0%)	468(100.0%)	468(100.0%)
SPICi	0(0.0%)	0(0.0%)	0(0.0%)	194(100.0%)	0(0.0%)	194(100.0%)
ClusterONE	0(0.0%)	0(0.0%)	0(0.0%)	268(100.0%)	0(0.0%)	268(100.0%)
PEWCC	121(6.12%)	133(6.73%)	153(7.74%)	1172(59.28%)	98(4.96%)	1677(84.83%)
WPNCA	25(3.35%)	24(3.21%)	28(3.75%)	625(83.67%)	12(1.61%)	714(95.58%)
WEC	0(0.0%)	0(0.0%)	0(0.0%)	408(100.0%)	0(0.0%)	408(100.0%)
ClusterEPs	N/A	N/A	N/A	N/A	N/A	N/A
ClusterSS	108(4.3%)	111(4.42%)	173(6.89%)	1425(56.75%)	175(6.97%)	1992(79.33%)
	126(5.31%)	92(3.88%)	233(9.83%)	1276(53.82%)	169(7.13%)	1896(79.97%)
SE-DMTG	41(2.91%)	40(2.84%)	57(4.05%)	1002(71.16%)	53(3.76%)	1193(84.73%)
MPC-C	91(4.23%)	70(3.25%)	160(7.43%)	1106(51.37%)	161(7.48%)	1588(73.76%)
GCC-v	5(2.03%)	13(5.28%)	54(21.95%)	115(46.75%)	29(11.79%)	216(87.8%)
MP-AHSA	0(0.0%)	0(0.0%)	0(0.0%)	535(100.0%)	0(0.0%)	**535(100.0%)**^{1st}

Note: the highest score of each row are shown in bold. N/A means that we fails to obtain the results under given code or software.
