# Supplementary material for: Detecting protein complexes with multiple properties by an adaptive harmony search algorithm
Source: BMC Bioinformatics. 2022 Oct 7;23:414. doi: 10.1186/s12859-022-04923-4 (PMC9541083; doi:10.1186/s12859-022-04923-4)
Supplement: Supplementary file 5 — Additional file 5. DIP PPI network. [file 12859_2022_4923_MOESM5_ESM.pdf]

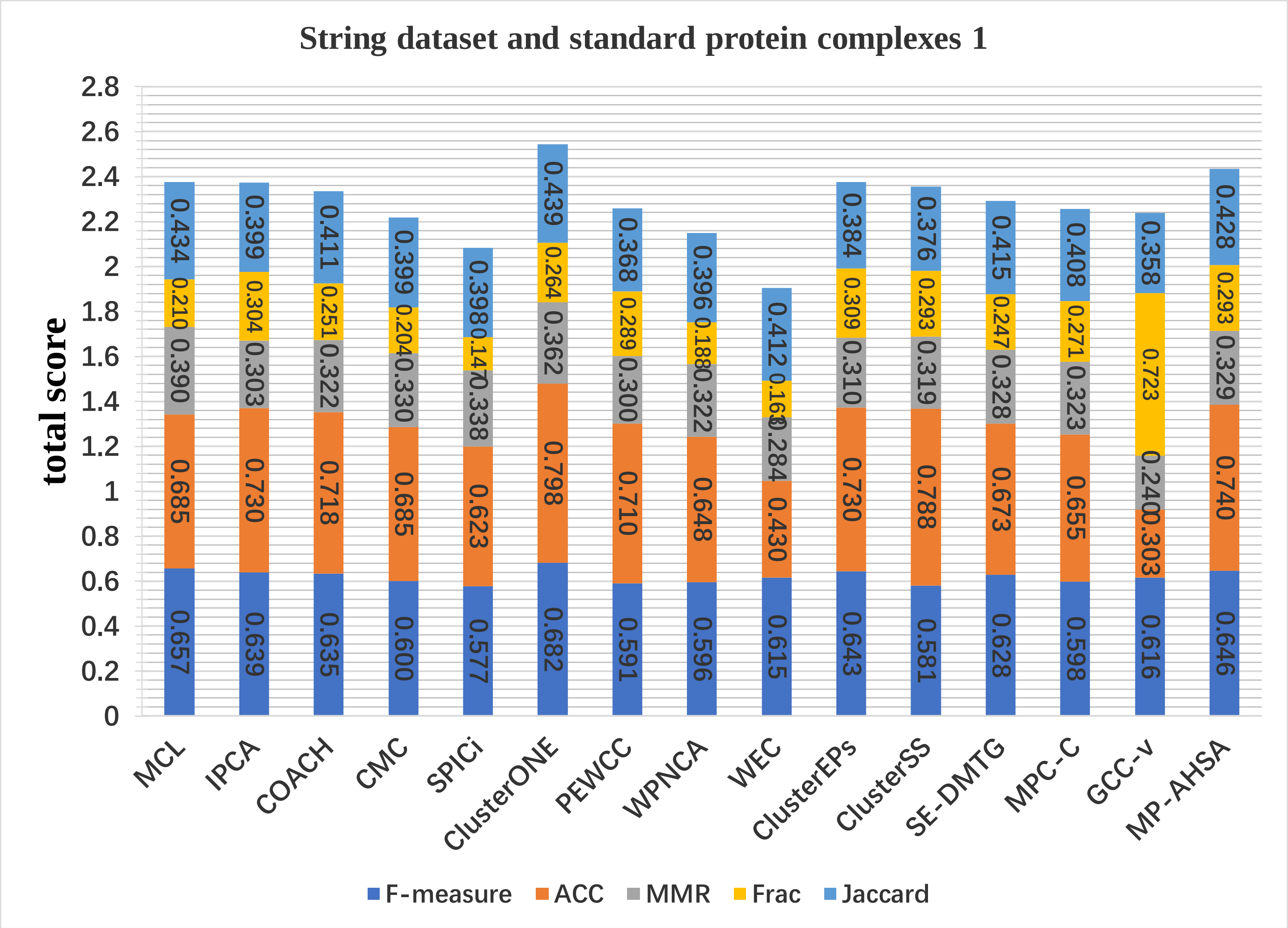
String dataset and standard protein complexes 1
total score
0
0.2
0.4
0.6
0.8
1
1.2
1.4
1.6
1.8
2
2.2
2.4
2.6
2.8
MCL 0.657 0.685 0.390 0.210 0.434
IPCA 0.639 0.730 0.303 0.304 0.399
COACH 0.635 0.718 0.322 0.251 0.411
CMC 0.600 0.685 0.330 0.204 0.399
SPICi 0.577 0.623 0.338 0.147 0.398
ClusterONE 0.682 0.798 0.362 0.264 0.439
PEWCC 0.591 0.710 0.300 0.289 0.368
WPNCA 0.596 0.648 0.322 0.188 0.396
WEC 0.615 0.430 0.284 0.412
ClusterEPs 0.643 0.730 0.310 0.309 0.384
ClusterSS 0.581 0.788 0.319 0.293 0.376
SE-DMTG 0.628 0.673 0.328 0.247 0.415
MPC-C 0.598 0.655 0.323 0.271 0.408
GCC-v 0.616 0.303 0.240 0.723 0.358
MP-AHSA 0.646 0.740 0.329 0.293 0.428
F-measure
ACC
MMR
Frac
Jaccard

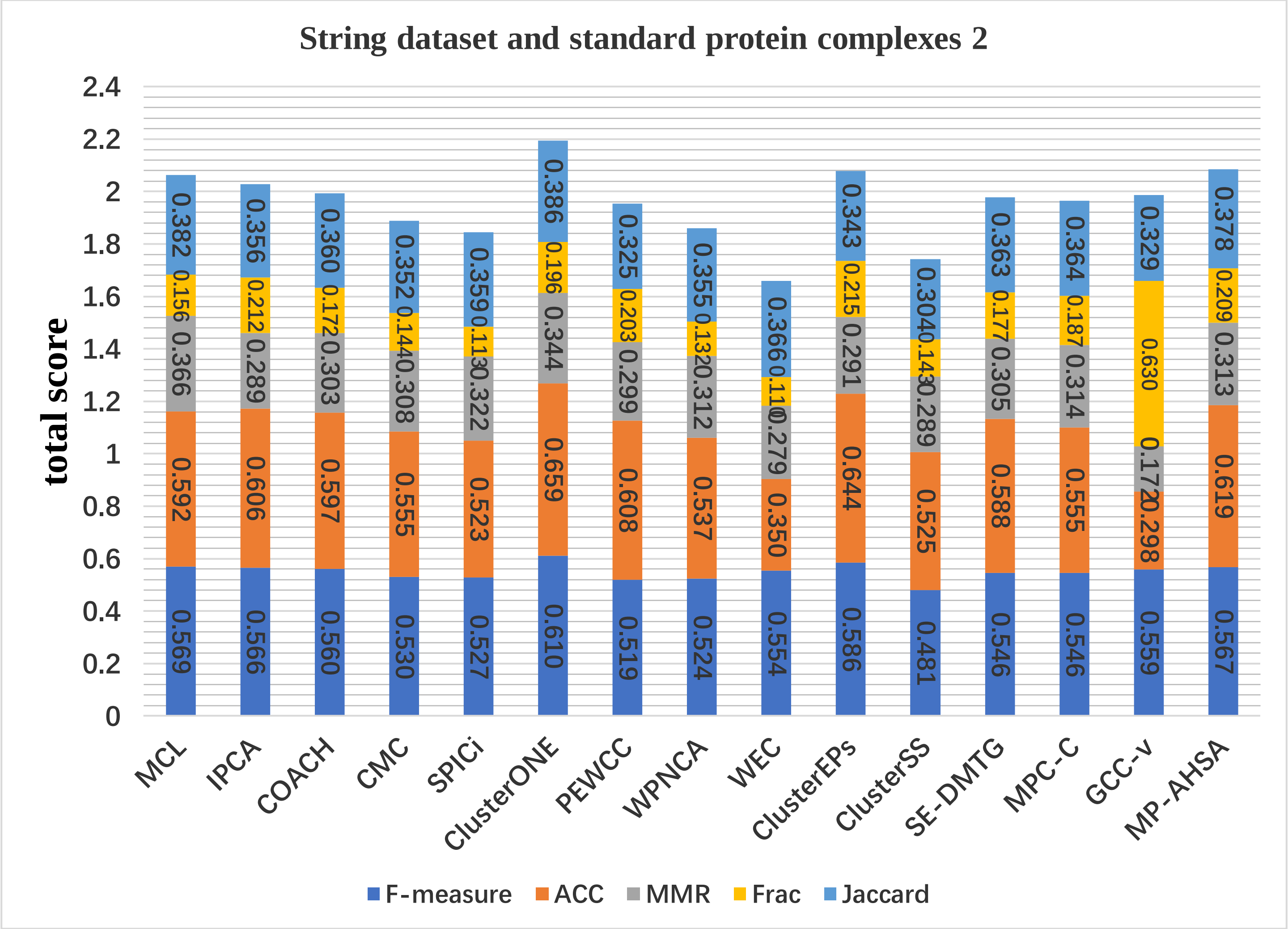
String dataset and standard protein complexes 2
total score
0
0.2
0.4
0.6
0.8
1
1.2
1.4
1.6
1.8
2
2.2
2.4
MCL 0.569 0.592 0.366 0.156 0.382
IPCA 0.566 0.606 0.289 0.212 0.356
COACH 0.560 0.597 0.303 0.172 0.360
CMC 0.530 0.555 0.308 0.144 0.352
SPICi 0.527 0.523 0.322 0.113 0.359
ClusterONE 0.610 0.659 0.344 0.196 0.386
PEWCC 0.519 0.608 0.299 0.203 0.325
WPNCA 0.524 0.537 0.312 0.132 0.355
WEC 0.554 0.350 0.279 0.110 0.366
ClusterEPs 0.586 0.644 0.291 0.215 0.343
ClusterSS 0.481 0.525 0.289 0.143 0.304
SE-DMTG 0.546 0.588 0.305 0.177 0.363
MPC-C 0.546 0.555 0.314 0.187 0.364
GCC-v 0.559 0.298 0.172 0.630 0.329
MP-AHSA 0.567 0.619 0.313 0.209 0.378
F-measure
ACC
MMR
Frac
Jaccard
