# Supplementary material for: Detecting protein complexes with multiple properties by an adaptive harmony search algorithm
Source: BMC Bioinformatics. 2022 Oct 7;23:414. doi: 10.1186/s12859-022-04923-4 (PMC9541083; doi:10.1186/s12859-022-04923-4)
Supplement: Supplementary file 7 — Additional file 7. Gene expression data. [file 12859_2022_4923_MOESM7_ESM.pdf]

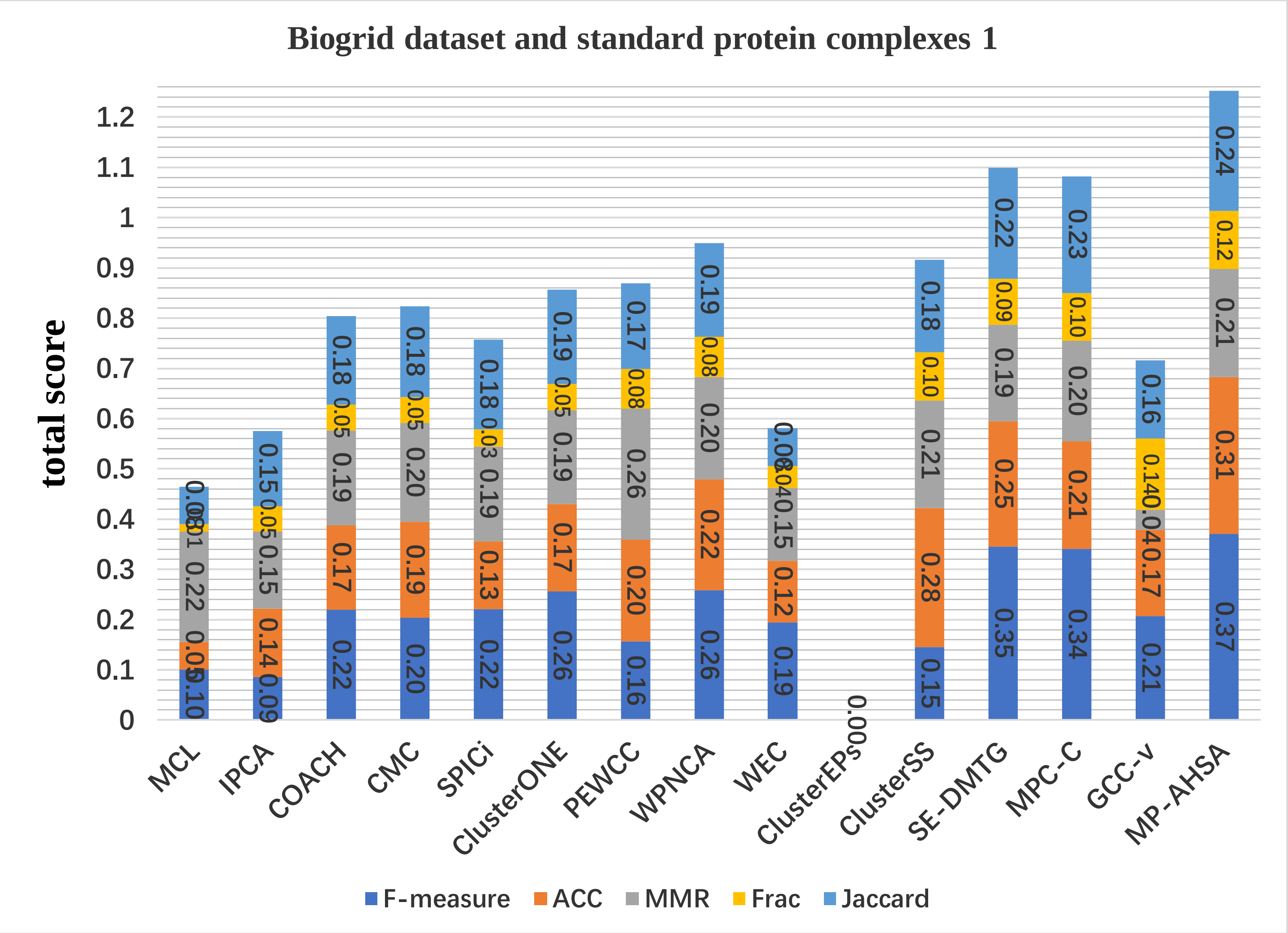
Biogrid dataset and standard protein complexes 1
total score
0
0.1
0.2
0.3
0.4
0.5
0.6
0.7
0.8
0.9
1
1.1
1.2
MCL
0.22
IPCA
0.09 0.14 0.15 0.05 0.15
COACH
0.22 0.17 0.19 0.05 0.18
CMC
0.20 0.19 0.20 0.05 0.18
SPICi
0.22 0.13 0.19 0.03 0.18
ClusterONE
0.26 0.17 0.19 0.05 0.19
PEWCC
0.16 0.20 0.26 0.08 0.17
WPNCA
0.26 0.22 0.20 0.08 0.19
WEC
0.19 0.12 0.15
ClusterEPs
0.00
ClusterSS
0.15 0.28 0.21 0.10 0.18
SE-DMTG
0.35 0.25 0.19 0.09 0.22
MPC-C
0.34 0.21 0.20 0.10 0.23
GCC-v
0.21 0.17 0.04 0.14 0.16
MP-AHSA
0.37 0.31 0.21 0.12 0.24
F-measure
ACC
MMR
Frac
Jaccard

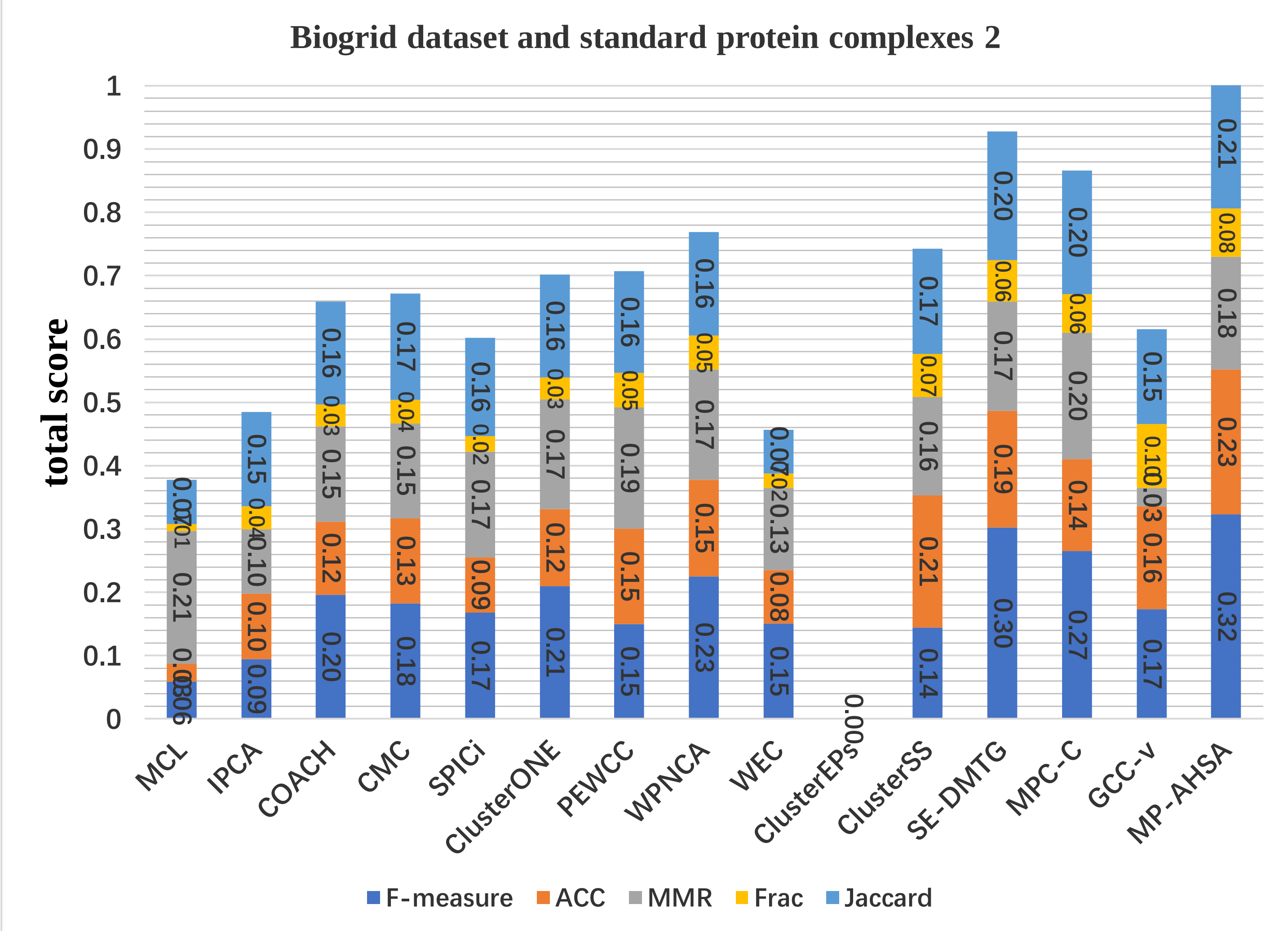
Biogrid dataset and standard protein complexes 2
total score
0
0.1
0.2
0.3
0.4
0.5
0.6
0.7
0.8
0.9
1
MCL
0.21
IPCA
0.09 0.10 0.10 0.04 0.15
COACH
0.20 0.12 0.15 0.03 0.16
CMC
0.18 0.13 0.15 0.04 0.17
SPICi
0.17 0.09 0.17 0.02 0.16
ClusterONE
0.21 0.12 0.17 0.03 0.16
PEWCC
0.15 0.15 0.19 0.05 0.16
WPNCA
0.23 0.15 0.17 0.05 0.16
WEC
0.15 0.08 0.13
ClusterEPs
0.00
ClusterSS
0.14 0.21 0.16 0.07 0.17
SE-DMTG
0.30 0.19 0.17 0.06 0.20
MPC-C
0.27 0.14 0.20 0.06 0.20
GCC-v
0.17 0.16 0.03 0.10 0.15
MP-AHSA
0.32 0.23 0.18 0.08 0.21
F-measure
ACC
MMR
Frac
Jaccard
